# Genome‐wide analysis of hybridization in wild boar populations reveals adaptive introgression from domestic pig

**DOI:** 10.1111/eva.13432

**Published:** 2022-07-02

**Authors:** Nicolas Mary, Nathalie Iannuccelli, Geoffrey Petit, Nathalie Bonnet, Alain Pinton, Harmonie Barasc, Amélie Faure, Anne Calgaro, Vladimir Grosbois, Bertrand Servin, Alain Ducos, Juliette Riquet

**Affiliations:** ^1^ GenPhySE, INRAE, ENVT Université de Toulouse Castanet Tolosan France; ^2^ ASTRE, CIRAD, INRAE Montpellier France

**Keywords:** admixture, feral pig, genetic monitoring, genotyping, population genetics, *Sus scrofa*, Wildlife management

## Abstract

The admixture of domestic pig into French wild boar populations has been monitored since the 1980s thanks to the existence of a cytogenetic difference between the two sub‐species. The number of chromosomes is 2*n* = 36 in wild boar and 2*n* = 38 in pig, respectively. This difference makes it possible to assign the “hybrid” status to wild boar individuals controlled with 37 or 38 chromosomes. However, it does not make it possible to determine the timing of the hybridization(s), nor to guarantee the absence of domestic admixture in an animal with 2*n* = 36 chromosomes. In order to analyze hybridization in greater detail and to avoid the inherent limitations of the cytogenetic approach, 362 wild boars (WB) recently collected in different French geographical areas and in different environments (farms, free ranging in protected or unprotected areas, animals with 2*n* = 36, 37 or 38 chromosomes) were genotyped on a 70K SNP chip. Principal component analyses allowed the identification of 13 “outliers” (3.6%), for which the proportion of the genome of “domestic” origin was greater than 40% (Admixture analyses). These animals were probably recent hybrids, having Asian domestic pig ancestry for most of them. For the remaining 349 animals studied, the proportion of the genome of “wild” origin varied between 83% and 100% (median: 94%). This proportion varied significantly depending on how the wild boar populations were managed. Local ancestry analyses revealed adaptive introgression from domestic pig, suggesting a critical role of genetic admixture in improving the fitness and population growth of WB. Overall, our results show that the methods used to monitor the domestic genetic contributions to wild boar populations should evolve in order to limit the level of admixture between the two gene pools.

## 1 INTRODUCTION

Domestic pigs (*Sus domesticus*, Erxleben, 1777) originated from the domestication of wild animals (wild boar: *Sus scrofa*, Linnaeus, 1758), which was initiated independently in Anatolia and in the Mekong Valley about 9000 years ago, from two different (European and Asian) wild boar populations that diverged one million years ago. In Europe, the domestication process has been going on for millennia and has involved regular gene flows between domestic flocks and populations of European wild boars (WB) different from the Anatolian population involved in the initial domestication process. The residual part of the genome of Near Eastern origin in modern European domestic pigs (DP) was estimated to vary between 0 and 4% (Frantz et al., 2015, 2019). The progressive development of pig farming and the selection of domestic populations have induced major genetic and phenotypic differences between DP and WB. These differences are also considerable between European and Asian DPs due to very different selection criteria between these two regions of the world and to differences between the wild populations from which the domestication took place.

The normal diploid number of chromosomes in the karyotype of DP is 2*n* = 38 (Gustavsson, 1988). In European WB populations, individuals with 2*n* = 36, 37 or 38 chromosomes have been described in France (Darre et al., 1992) and in the Iberian Peninsula (Nombela et al., 1990), as well as in some Northern and Eastern European countries (Aravena & Skewes, 2007). The only difference between the three karyotypes is the existence of a Robertsonian translocation between chromosomes 15 and 17: rob(15;17). In DPs (2*n* = 38), these two pairs of chromosomes are independent and acrocentric. In WBs (2*n* = 36), the chromosomes of these two pairs are fused to form a single pair of submetacentric rob(15;17) chromosomes. Large‐scale cytogenetic monitoring carried out between 1981 and 1991 in France revealed a significant variation in the number of chromosomes per individual depending on the nature of the WB populations considered (wild or farmed) and depending on whether populations were managed in nature reserves or in hunting federations (Darre et al., 1992). The percentage of hybrid individuals (with 2*n* = 37 or 38 chromosomes) in WB farms ranged from 0 (in one‐third of the farms) to 85%, and was about 20% in wild populations managed by hunting federations (Darre et al., 1992). On the contrary, of the 204 analyses carried out in wild populations from five nature reserves managed by government agencies, only two boars with 2*n* = 37 chromosomes (less than 1%) were detected (Darre et al., 1992). As a result of this and other studies reviewed in Aravena and Skewes (2007), the normal diploid number of chromosomes in Western European WBs has been set at 2*n* = 36. In cytogenetic terms, a WB with 2*n* = 36 chromosomes is, therefore, homozygous for the Robertsonian translocation rob(15;17) and cannot be a first‐generation hybrid. Indeed, mating a DP (2*n* = 38) with a WB (2*n* = 36) produces hybrid individuals with 37 chromosomes, which inherit one chromosome from each of pairs 15 and 17 from their DP parent, and one fused chromosome rob(15;17) from their WB parent (these individuals can be described as heterozygous for the translocation). These 2*n* = 37‐chromosome animals can be mated: (1) to other 37‐chromosome animals (expected to produce 25% offspring with 36 chromosomes, 50% with 37 chromosomes and 25% with 38 chromosomes); (2) to 36‐chromosome animals (expected to produce 50% offspring with 36 chromosomes and 50% with 37 chromosomes); or (3) to 38‐chromosome animals (expected to produce 50% offspring with 37 chromosomes and 50% with 38 chromosomes).

During the 18th and 19th centuries, the abundance and distribution of WB populations were considerably reduced in Europe. However, the interest shown by hunters for this species subsequently led to restoration attempts, including reintroductions of captive‐bred individuals into wild populations (Veličković et al., 2016; Yamamoto, 2017). Since the 1970s, WB populations have undergone very large and uncontrolled demographic growth (Albrycht et al., 2016; Massei et al., 2015) and this species is considered invasive in many regions (Barrios‐Garcia & Ballari, 2012). In France, data from the National Hunting and Wildlife Agency showed that the number of WBs killed by hunters has increased by 45% in 10 years and by 134% in 20 years (323,000 in 1997, 552,000 in 2007 and 756,000 in 2017). Several environmental changes have contributed to this very significant expansion, such as the evolution and intensification of crops, the reduction in the number of predators, and the global increase in average temperatures (Root et al., 2003). Other human activities such as WB breeding for hunting activities and (voluntary or involuntary) hybridization with DPs may also explain this expansion (Khederzadeh et al., 2019). Uncontrolled hybridizations may come from outdoor pig farms, whose number, although small (2.7% of sows and 1.6% of pigs are raised outdoors in France), has moderately but steadily increased since 2010 in some French areas. Another source of hybridization is the increased number of pigs raised as pets, especially Vietnamese pot‐bellied pigs (Gillespie et al., 2015; Østevik et al., 2012; Tynes, 2001). These pets have recently experienced a decline in popularity, explaining the increase in the number of abandonments (Delibes‐Mateo & Delibes, 2013).

Increased contacts between WBs and DPs lead to significant sanitary and safety risks for the commercial pig farms and the populations of the regions concerned (Hars & Rossi, 2010). The recent spread of African swine fever in northern Europe, for example, is currently posing very serious threats to the European pig industry (Blome et al., 2020). Increased contacts between the domestic and wild compartments also threaten the preservation of the gene pool of wild species (Wayne & Shaffer, 2016). In order to limit these risks and to discourage some of the practices that can cause them, a monitoring program for French WB populations was set up in France in the 1980s. This program, based on the cytogenetic difference between WBs and DPs, imposes quite strong constraints for breeders. “Category A” WB farms, which produce animals that can be released into the wild, can only raise animals of the *Sus scrofa* species with 2*n* = 36 chromosomes (Charlez, 2010). This program made it mandatory to carry out a cytogenetic analysis of any animal entering these farms and, at the same time, made it possible to assess the chromosomal status of wild populations and its evolution from animals captured in the wild (Darre et al., 1992).

While the implementation of this program has probably limited the frequency of voluntary hybridizations, chromosome counting by cytogenetic techniques used so far to carry out the controls has considerable limitations. The first one is that it does not guarantee that an individual with 36 chromosomes is not the product of hybridization(s): for example, an animal with 36 chromosomes can be the result of a mating between two individuals with 37 chromosomes. The other limitation is that it does not allow, in the case of an individual with 37 chromosomes, for example, to date the event (or events) of hybridization that is (or are) at the origin of this animal, nor to quantify the proportion of its genome of DP origin. Another limitation of cytogenetic analyses is the necessity of carrying out cell cultures and, therefore, of having samples taken from living (or recently dead) animals in conditions that avoid the contamination of cell cultures, conditions that are very difficult to satisfy when biological samples are taken from wild animals. A possible approach to overcome these limitations is the genotyping of animals for molecular markers distributed throughout the genome (Goedbloed, Megens, et al., 2013).

The present study was carried out with two main objectives. The first one was to analyze the results of the cytogenetic control program conducted in France between 2008 and 2020 in light of genome‐wide genotyping data and to study the possibility and relevance of implementing a new method for assessing hybridization using genome‐wide SNP genotyping. The second objective was to update our knowledge about the level of introgression in different French WB populations, using both cytogenetic and molecular data.

Three hundred and sixty‐two WBs were genotyped using a 70 K SNP pig chip. These 362 animals with different chromosomal status (2*n* = 36, 37 or 38) were sampled from different types of management units and from different French regions. Our results confirm the limitations of the cytogenetic analyses carried out so far and show that the WB population monitoring program would be greatly improved by genotyping. We also show that 13 individuals (out of 362 WB studied, i.e., 3.6%) were likely recent hybrids, while 210 (out of 349, i.e., 60%) showed traces of introgression. This result is an indication of the threat to biodiversity posed by admixture and suggests that new preservation measures should be considered to improve its management.

## 2 MATERIAL AND METHODS

### 2.1 Selection of the analyzed samples

#### 2.1.1 WB samples used in the study

A total of 362 WBs were genotyped in this study (Table 1).

**TABLE 1:** Different categories of WB sampled

Categories	Genotyped	Karyotyped	2*n* = 36	2*n* = 37	2*n* = 38
WB_Preserved^a^	56	56	56	0	0
WB_Deux‐Sèvres^b^	28	28	13	14	1
WB_Ardèche^c^	82	3	3	0	0
Other WBs	196	196	131	56	9
Total	362	283	203	70	10

*Note*: The second column indicates the number of individuals genotyped. Among them, the third column indicates the number of individuals karyotyped. The 2*n* = 36, 2*n* = 37, and 2*n* = 38 columns indicate the number of individuals with the corresponding karyotype.

^a^ WBs from management units (farms or parks) where admixture was considered unlikely based on 12 years of cytogenetic analyses.

^b^ Free‐ranging WBs from protected areas (nature reserves).

^c^ Free‐ranging WBs from an unprotected area.

Of these, 280 underwent cytogenetic analysis between 2017 and 2019 (blood samples were stored specifically for the present study). These 280 animals were selected to be representative of the diversity of the management units that sent samples to the laboratory during this period (WB farms, hunting parks and nature reserves). Among them, we identified seven management units that had requested a relatively large number of analyses over the last 12 years (between 35 and 112) and exhibited very high rates of 2*n* = 36 animals (>97%; Table S1). Animals from these units with very few or no “cytogenetical hybrids” were considered as being preserved from hybridization (they were the ones for which the risk of hybridization seemed the lowest a priori). Fifty‐six samples (out of 280) belonged to this category (referred to as WB_Preserved; Table 1). Twenty‐eight additional samples (out of 280) came from individuals caught in a nature reserve in Western France managed by a government agency (the administrative French subdivision, Deux‐Sèvres, hereafter referred to as WB_Deux‐Sèvres; Table 1) with high rates (>40%) of individuals with 37 or 38 chromosomes since the 2000s (much lower rates before that date). The remaining individuals (196 out of 280) were chosen in order to represent the different French geographical areas.

Eighty‐two additional WB samples (79 skin biopsies plus three blood samples taken by hunters between 2013 and 2017) came from an area that is not subject to particular protection measures (the administrative French subdivision, Ardèche, hereafter referred to as WB_Ardèche; Table 1). These animals were initially sampled for another study (Petit et al., 2020). Analysis of the chromosomal status (2*n* = 36, 37 or 38 chromosomes) could only be performed for three animals out of 82 (those for which blood samples were available).

The overall geographical distribution of the WBs sampled and analyzed in this study and their chromosomal status are presented in Figure S1.

### 2.2 DP samples used in the study

As already observed in Spain (Delibes‐Mateo & Delibes, 2013), the hybridization of French WBs with “pot‐bellied pigs” (or “Vietnamese pot‐bellied pigs,” DP of Asian origin used as pets) has been suspected on various occasions (animals released into the wild by their owners; Petit, personal communication). Two such animals were, therefore, added to our collection (individuals of probable Asian ancestry but whose precise genetic origins were undetermined). Biological samples (skin biopsy and blood) were taken from these animals during a routine surgical castration performed at the National Veterinary School of Toulouse at the request of the owners.

Genotyping data for DP performed in previous projects (Mercat et al., 2020; Muñoz et al., 2019) using the PorcineSNP60 (v1 and v2; Illumina Inc.) or GGP70K chips were also used in the present study. A total of ten pig breeds were included (Table S2). These included five commercial breeds, four of European origin (Duroc, Landrace, Large White and Piétrain) and one of Asian origin (Meishan), as well as five local French breeds (Basque, Bayeux, Gascon, Limousin, Porc Blanc de l'Ouest). A principal component analysis (PCA) was performed independently for each DP breed using the snpgdsPCA function of the SNPRelate v1.18.1 R package (Zheng et al., 2012) in order to identify the few individuals that strongly differed from their breed of origin. To guarantee the robustness of subsequent analyses, these individuals, potentially corresponding to identification errors or resulting from hybridization events (between different pig breeds or with WB), were removed. The number of animals genotyped in the commercial breeds was very large (several thousand). To balance the sample sizes between the different breeds studied and to limit the computation times, we selected 100 individuals for each of the commercial breeds (Table S2). To maximize the genetic diversity within the samples studied (rather than making a simple random selection of 100 individuals per commercial breed), we applied IBS (Identity by State) thresholds (different thresholds for each of the four commercial breeds studied) beyond which we excluded one individual from each pairwise comparison (within each pair, the individual with the best call rate was selected). These analyses were performed with the R SNPRelate package.

None of the animals used in our study were bred, killed, or captured specifically for the needs of our project, which therefore did not require explicit authorization (in accordance with European Directive 2010/63/EU).

### 2.3 Cytogenetic monitoring of French wild boar populations

Mitotic chromosomes were prepared from nonsynchronized cultures of peripheral blood lymphocytes collected on heparinized tubes. Whole blood (1 ml) was cultured for 72 h in a medium consisting of 9 ml RPMI (Gibco), 20% fetal bovine serum, and 500 IU Heparin (Sanofi), and stimulated with 0.2 ml pokeweed mitogen (Gibco). Hypotonic treatment (10 ml 1/6 calf serum) was followed by prefixation and fixation in ethanol: acetic acid (3:1). Chromosome preparations were spread on cold wet slides and air‐dried. Slides were stained with 3% Giemsa solution. For each individual, the number of chromosomes of at least ten cells was counted.

### 2.4 DNA extraction and genotyping

The DNA of 362 WBs and two domestic “Vietnamese” pigs was extracted from the blood samples using the Blood DNA Isolation kit (Norgen) and a classical protocol (lysis with proteinase K and ethanol precipitation) for skin biopsies. Tubes containing 4 μl of gDNA diluted to 50 ng/μl were prepared for genotyping, performed on the CRCT's Genomics and Transcriptomics platform (www.poletechno‐crct.inserm.fr) using a GeneSeek Genomic Profiler chip (GGP70 K HD Porcine, Illumina Inc.) comprising 68,516 SNPs. All the boars had a call rate >0.90 with an average of 0.93. The complete genotypes dataset produced in this paper is described in a data paper (Iannuccelli et al., 2022).

### 2.5 Genetic structure of populations and analysis of pig × wild boar hybridization

The 40,241 SNPs common to the three genotyping chips, located on the autosomes and for which the rates of missing genotypes were less than 10%, were retained for further analyses. Since hybridization (as well as other evolutionary forces such as selection or genetic drift) was likely to induce a deviation from the Hardy–Weinberg equilibrium (HWE), and insofar as we suspected the presence of introgressed individuals in our WB sample, this HWE criterion was not used for variant filtering.

A PCA was first performed using the snpgdsPCA function of the SNPRelate R package by considering all of the genotyped animals (DPs as well as WBs) in order to define the main groups (clusters) of animals. WBs that do not belong to their cluster (animals hereafter referred as WB_Outliers, potentially resulting from recent hybridization event(s)) were identified using a Bayesian method implemented in the Mclust function of the mclust R package v5.4.7 (Scrucca et al., 2016) using the values of components 1 and 2 of the PCA.

In a second step, the Bayesian clustering method implemented in Admixture software v1.3.0 (Alexander et al., 2009) was used to quantify the proportions of the different possible ancestral origins in the genome of each individual. An unsupervised analysis (without prior knowledge about the different ancestral populations) was performed using the software's default settings for *K* values ranging from 2 to 25. The optimal *K* value was estimated using a cross‐validation procedure for each *K* value. The value retained (*K* = 11) corresponds to the value for which the cross‐validation curve reached a plateau. The R Pophelper package v2.3.1 (Francis, 2017) and the Python Pong package v1.4.9 (Behr et al., 2016) were used to visualize the results of these analyses. Standard errors (SE) of ancestral origin estimates (the proportion of each individual's genome originating from each of the 11 clusters previously defined) were calculated using a random sampling procedure (bootstrap, 200 replications). These SEs were used to determine the confidence intervals at the 95% threshold of the estimated values (±1.96*SE). The proportions of different ancestral origins were compared between populations (or groups) of WBs using the Kruskal–Wallis test. To categorize animals as “unadmixed WBs,” we determined whether the 95% confidence intervals for the Admixture *Q* scores overlapped 0.99 of WB ancestry (only individuals with a “significant” fraction of their genome of domestic origin, i.e., greater than 1%, were considered as introgressed WBs).

### 2.6 Local ancestry inference

Local ancestry analyses along all autosomes were carried out with two main objectives. The first one was to detect and characterize introgression from DP into WB populations. The second objective was to assess the relevance of a possible in silico prediction of the chromosomal status of WBs (number of chromosomes: 2*n* = 36, 37 or 38) using genotyping data.

Local ancestries along chromosomes were inferred using two different approaches: ELAI v1.0 (Efficient Local Ancestry Inference), which condenses and groups haplotypes into different groups and assigns each local haplotype probabilistically into groups (Guan, 2014), and LAMP v2.4 (Local Ancestry in adMixed Populations, Sankararaman et al., 2008), a less efficient but computationally faster approach.

In order to predict the chromosomal status of WBs (2*n* = 36, 37 or 38) using genotyping data with reasonable computing time, we used LAMP around the fusion break point to estimate, for each possible ancestry, the allelic proportions (0, 0.5 or 1) for each individual and each SNP. We, therefore, considered that a WB should have a 2*n* = 36 karyotype if the two alleles of the first SNPs on both sides of the centromere of the rob(15;17) have a WB ancestry, 2*n* = 37 if one of the two alleles has a DP ancestry, and 2*n* = 38 if the two alleles of the first SNPs on either side of the centromere have a DP ancestry (see explanations given in Figure S2).

To analyze the ancestry along chromosomes 15 and 17, the latter were fused to create a rob(15;17) reference sequence in silico. For that purpose, chromosome maps were fused by their centromeres and the SNPs' positions were reordered accordingly (from the telomeric part of SSC17 to the telomeric part of SSC15, assuming a total length of 63,494,081 nucleotides for chromosome 17; the first nucleotide on the q‐arm of the rob(15;17) was, therefore, nucleotide 63,494,081 + 1).

To differentiate between wild and domestic origins at each genomic position, three training samples were used with each program (ELAI and LAMP): one for WBs and two for DPs. The first DP population was made up of animals belonging to breeds of European origin, without the Duroc breed (*n* = 614; the Duroc breed was eliminated since the PCA showed that it does not belong to the same cluster as the other European breeds—see below), while the second DP population was composed of the Meishan breed, of Asian origin (*n* = 35). As we used ELAI and LAMP with different objectives (detection of WB ancestry along all autosomes, and in silico prediction of the chromosomal status, respectively), the WB training samples used were different between the two programs. For ELAI, the WB training sample was composed of animals defined as “unadmixed” (*n* = 139) following admixture analysis. To improve the in silico prediction of chromosomal status performed by LAMP, we used all WBs with a 2*n* = 36 karyotype (WB_Outliers excluded) as a WB training sample (*n* = 201).

Autosomal SNP was filtered with MAF > 0.05 and missing rate <0.05. ELAI was run with 30 steps in the expectation–maximization (EM) run. The values for “upper‐layer clusters” and “lower‐layer clusters” were set to 3 and 15, respectively, as recommended by the author (Guan, 2014). LAMP parameters were adjusted to delete SNP in linkage disequilibrium (the *r* ^2^ cutoff was set to 0.1: all but one of the SNPs in LD are retained for the ancestry estimation). We used a recombination rate of 1 × 10^−8^ and a fraction of overlap between adjacent windows of 80% (offset parameter = 0.2). Mixture proportions of 0.30 (WBs), 0.66 (European DPs), and 0.04 (Asian DPs) were assumed, which corresponded to the ancestry proportions estimated using Admixture software for *K* = 11. The number of admixture generations was set to 25 for both applications. This number of generations allows us to detect the modern history of hybridization between the two subspecies rather than the gene flow that has been taking place throughout the domestication process. In addition, Guan (2014) demonstrated that ELAI is robust to the number of admixture generations, which mainly affects the smoothness of the local ancestry inference.

## 3 RESULTS

### 3.1 Cytogenetic monitoring of French wild boar populations

The number of cytogenetic analyses of WBs carried out annually in France between 2008 and 2020 varied between 160 and 576, with an average of 376. The decrease in the number of analyses has been fairly clear since 2018 (Figure S3). The overall rate of “cytogenetical hybrids” (animals with 37 or 38 chromosomes, which may be the products of recent or ancient hybridization event(s)), calculated from the results obtained for the 4894 individuals analyzed in our laboratory during this period, was 15.3% (13.0% of 2*n* = 37 and 2.3% of 2*n* = 38 animals). This rate varied quite strongly from 1 year to the next (Figure S3), due to sampling fluctuations. The management units that requested analyses were not the same from 1 year to the next, and hybrid rates varied greatly from one unit to the other (from 0% to 40% for management units having performed at least 25 analyses between 2008 and 2020). Given these large sampling variations, the apparent decrease in the hybrid rate observed since 2017 should be considered with caution.

### 3.2 Principal component analyses (PCA)

**FIGURE 1:**
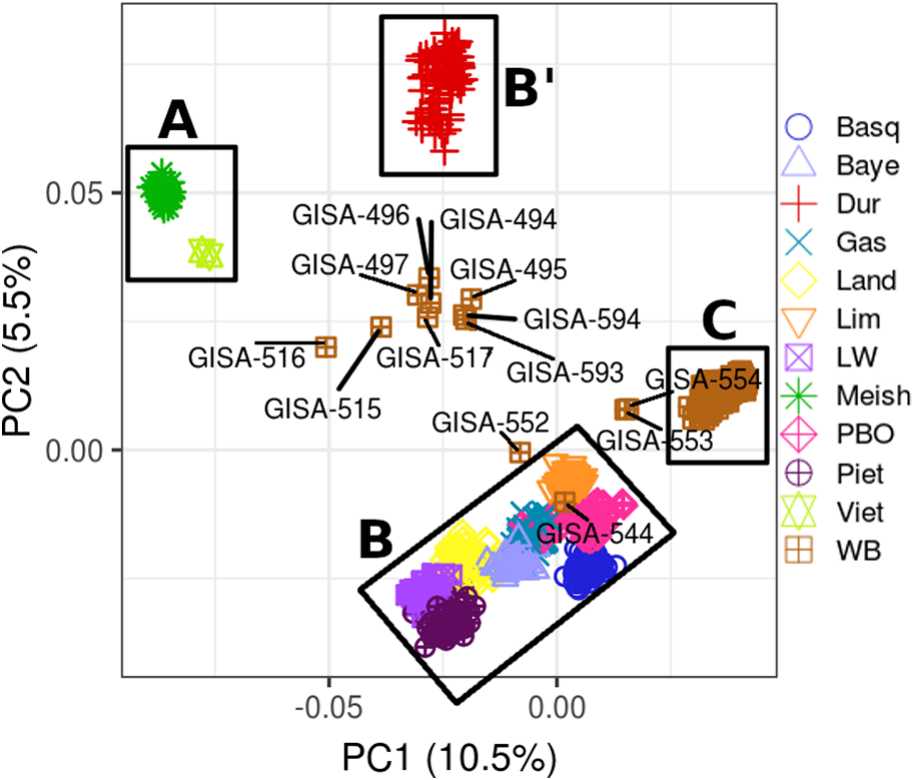
Population structure defined with PCA of 714 pigs from nine European breeds, 37 pigs from two Asian breeds, and 362 French WB. The first (PC1) and second (PC2) principal components are shown. The WBs outside the WB cluster (WB_Outliers) are represented with their respective identification numbers (GISA‐xxx). Letters A, B/B′ and C represent the Asian pig breeds, European pig breeds, and wild boar groups, respectively

The first and second axes of the PCA performed using all genotyping data (WBs and DPs of local as well as commercial breeds) explained 10.5% and 5.5% of the total variance, respectively (Figure 1). The explained variance gain became very small from principal component 11 onwards (Figure S4), which corresponds to the number of populations analyzed (considering Meishan and “Vietnamese” type pigs as one single cluster of “Asian” origin). The first principal component allowed the separation of three groups of animals. The first one (noted A in Figure 1) consisted of individuals of Asian pig breeds; the second group was composed of animals of European pig breeds (B and B′), while the last group was composed of WBs only (C). The second principal component also made it possible to separate the WBs from the Asian and European DP breeds, as well as to distinguish the Duroc DP breed, which formed a group that was distinct from the other European pig breeds (confirmation of previous results: see, e.g., Lee et al., 2020).

The first two principal components were also used to specifically explore the WB population in order to identify individuals outside the group. Thirteen individuals (out of 362, i.e., 3.6%) from six different management units were identified that did not belong to the WB cluster. None of these was detected in the free‐ranging populations. Those individuals, hereafter referred to as “WB_outliers,” were not considered as WBs and were, therefore, analyzed separately.

### 3.3 Admixture analyses

Unsupervised admixture analysis was performed using an increasing number of populations (*K* parameter varying from 2 to 25; Figure S5). For *K* = 2, WBs could be distinguished from Asian pigs. For *K* = 3, European and Asian DP breeds, as well as WBs, could be distinguished. From *K* = 4 to *K* = 11, the different European breeds appear as different gene pools, one after the other. From *K* = 12 onwards, substructures appear within certain populations (gene pools), for example, between different groups of WBs (*K* = 12 and 13), or between different groups (lines) within the Duroc breed (*K* = 14). The lowest cross‐validation value was obtained for *K* = 25 (Figure S6). In that situation, substructures per farm (related animals) and/or geographical regions appeared for the majority of the gene pools studied. The cross‐validation value reached a plateau at *K* = 11. This value was consistent with the number of clusters resulting from the PCA. Moreover, a very strong correlation was observed for WBs between the first component of PCA and WB ancestry estimates for *K* = 11 (without WB_outliers: *r* = 0.935, *p* < 0.001). We, therefore, considered that the first level of structuring of the populations analyzed was 11. This value allowed us to define a cluster (a dominant color) for each genotype except “Vietnamese” pigs. The very low number of animals of this type genotyped in our study (*n* = 2) did not allow us to attribute a specific cluster to them. However, the ancestral origin of the genomes of these two “Vietnamese” animals was quite close to that of the Meishan animals, confirming their Asian origin.

#### 3.3.1 Admixture analyses for WB (animals assigned to the WB_cluster)

**FIGURE 2:**
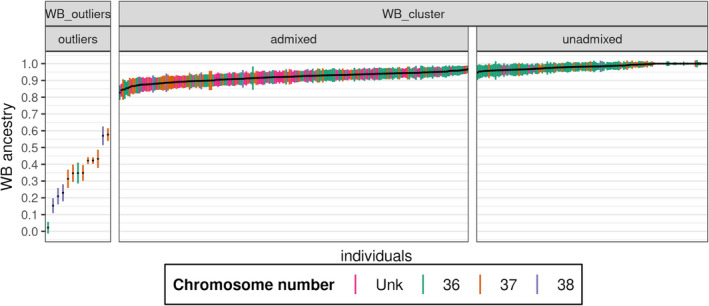
Estimates of WB ancestry and 95% confidence intervals (CI) for all 362 individuals. Individuals are arranged by *Q* score following admixture analysis for *k* = 11. The colors represent the chromosome number of each individual. The first vertical line (on the left side) separates the outliers from animals belonging to the WB cluster (based on PCA). Among the WB_cluster, animals were considered as “unadmixed” if the 95% CI overlapped 0.99 (proportion of WB ancestry)

For the 349 individuals belonging to the WB_cluster (outliers excluded), the proportion of the genome of WB origin (Figure 2) varied between 0.83 and 1 (mean and median equal to 0.94). Within the WB_cluster, 210 individuals (60%) were considered as “admixed” (Figure 2), and 139 (40%) as “unadmixed” (i.e., animals for which the 95% confidence intervals for the Admixture Q scores overlapped 0.99). However, the distinction between these two categories is arbitrary, and some individuals with 37 or even 38 chromosomes were considered as unadmixed (Figure 2), which may seem counterintuitive at first glance. As shown in Figure S7a, the proportion of the genome of WB origin was significantly higher for individuals with 2*n* = 36 chromosomes (median: 0.96) than for individuals with 2*n* = 37 or 38 chromosomes (medians 0.93 and 0.90, respectively).

For the 56 individuals belonging to the seven management units where admixture was considered unlikely based on cytogenetic results (WB_Preserved), the proportion of the genome of “wild” origin was 0.98 (median value; Figure S7b). This proportion was also very high (median value: 0.99) in WB_Deux_Sèvres (individuals sampled in a nature reserve), although the number of animals with 2*n* = 37 and 38 chromosomes was quite high in that population (Figure S1). It was significantly lower (median value: 0.92) for WB_Ardèche (animals collected in an unprotected area; Figure S7b).

#### 3.3.2 Admixture analyses for the outliers (animals assigned to WB_outliers)

**FIGURE 3:**
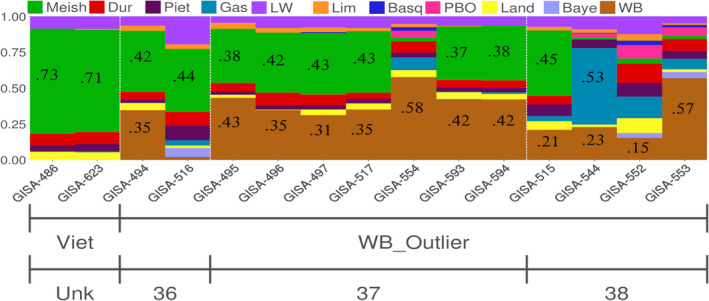
Admixture analysis (*K* = 11) of wild boar outliers (+ “Vietnamese” DP on the left of the figure). The different colors represent the dominant ancestral proportions of the different breeds (gene pools) considered in this study

Analyses performed with *K* = 11 showed DP origins greater than 40% for all of the 13 WB_outliers (Figure 3), suggesting that these animals were recent hybrids. The majority of these outliers (9/13) had a significant part of their genome (>37%) of “Asian” origin (green in Figure 3).

The genomic compositions of the three other outliers (552, 553, and 554) presented comparable characteristics: almost no Asian origin, the presence of a mosaic of European origins (which was different however from one individual to another). This suggests hybridizations with several breeds of European origin or with a European breed other than the ones analyzed in this study.

One hybrid (outlier 544) had 53% (±3%) of its genome of Gascon origin (local breed with a large proportion of outdoor breeding farms).

Finally, and quite surprisingly, outlier 516 had only 2% (±2%) of its genome of WB origin. This individual presented a 2*n* = 36 chromosome karyotype, which was confirmed in silico (see the “*in silico* prediction of chromosomal status” section below), suggesting that it was the product of numerous backcrosses with DP breeds.

### 3.4 Local ancestry inferences

#### 3.4.1 Distribution of the WB ancestry along the autosomes (ELAI)

To look for signals of recent introgression in WB populations, we ran a three‐way admixture inference with ELAI for all animals belonging to the WB_Cluster. Genome‐wide WB ancestry estimates per individual obtained with ELAI, on the one hand, and unsupervised Admixture, on the other hand, were highly correlated (*r* = 0.916, *p* < 0.001), which suggests that the choice of ELAI parameters was appropriate.

**FIGURE 4:**
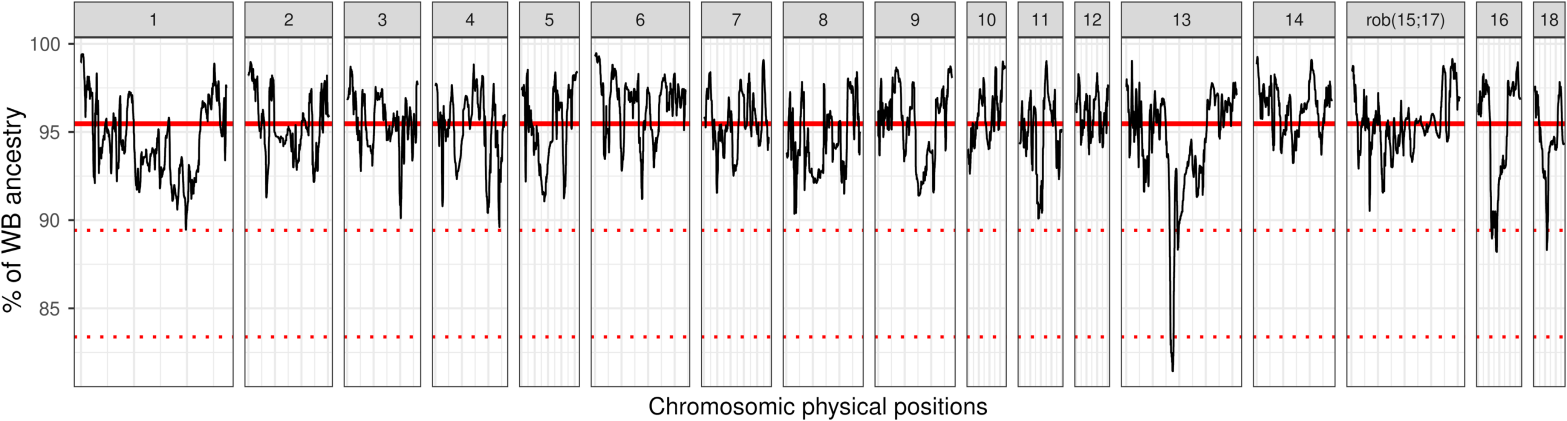
Average wild boar ancestry estimated over the 349 WB (without outliers) using ELAI, for each position of each autosome. The bold red line represents the mean ancestry, and dotted red lines represent a deviation of three SD and six SD from the mean

To detect genomic regions with unusually high or low levels of WB ancestry, the proportion of WB ancestry was averaged over the 349 WBs at each genomic position. Overall (on a genome‐wide basis), the proportion of WB ancestry was 95.5 ± 2%. The distribution of the WB ancestry along the autosomes (Figure 4) revealed unusually low levels (and a corresponding increase in DP ancestry) on chromosomes 13, 16, and 18. The strongest introgression signal, which reached a maximum of 19% of DP ancestry, was observed on chromosome 13. To investigate the region concerned in more detail (see the Discussion section), we defined a genomic interval of 5.6 Mb on this chromosome (from 83.46 to 89.08 Mb) for which the average proportion of WB origin was six standard deviations lower than the mean.

To analyze the origin of chromosomes 15 and 17 in WBs exhibiting 2*n* = 37 (WB_37) or 38 chromosomes (WB_38), we specifically looked at the proportion of WB ancestry along the reconstituted rob(15;17) chromosome according to the number of chromosomes established using cytogenetic analyses (Figure S8). The ancestral origin of the 201 WBs with 2*n* = 36 chromosomes is overwhelmingly “wild” all along the reconstituted rob(15;17). For the 63 WBs with 2*n* = 37 chromosomes and the six WBs with 2*n* = 38 chromosomes, we observed the lowest level of WB ancestry (and a corresponding increase in DP ancestry) on the pericentromeric region of this chromosome.

#### 3.4.2 In silico prediction of the chromosomal status (LAMP)

In light of the results obtained with ELAI regarding the centromeric region of rob(15;17), we performed complementary analyses using LAMP. The chromosomal status of each WB with a known number of chromosomes (established using cytogenetic techniques; *n* = 283; Table 1) was inferred in silico according to the ancestry origins of the closest SNPs on both sides of the centromere of the rob(15;17) and then compared with the actual numbers of chromosomes. The overall concordance rate (CR) for all 283 WBs was quite high (94.7%). As shown in Table 2, predictions were very accurate for individuals with 2*n* = 36 and 2*n* = 38 chromosomes (CRs equal to 99.5% and 100%, respectively) but less effective for individuals with 2*n* = 37 chromosomes (CR = 80.0%).

**TABLE 2:** Comparison of the actual numbers of chromosomes (in vitro, established using cytogenetic techniques) with the ones predicted in silico (LAMP software)

	In vitro (real)		
	2*n* = 36 (*N* = 203)	2*n* = 37 (*N* = 70)	2*n* = 38 (*N* = 10)
In silico (prediction)			
2*n* = 36	99.5% (202/203)	20.0% (14/70)	0% (0/10)
2*n* = 37	0.5% (1/203)	80% (56/70)	0% (0/10)
2*n* = 38	0% (0/203)	0% (0/70)	100% (10/10)

## 4 DISCUSSION

The genetic composition of wild and farmed populations of WBs, in France and more widely in Europe, has been of interest to scientists, wildlife managers, and public authorities for many years because of the significant ecological, economic, and health consequences that hybridization with DPs is likely to induce. Distinguishing between unadmixed WBs and hybridized individuals can be achieved using different approaches. Basing the distinction on morphological criteria only can be difficult and seems rather irrelevant (Aravena & Skewes, 2007). The use of genetic analysis is potentially more informative. Large‐scale retrospective studies carried out to date in France (Darre et al., 1992; Ducos et al., 2008) relied on cytogenetic analyses only, which have considerable limitations. The first one is that cytogenetic analysis does not guarantee that an individual with 36 chromosomes is not the product of past hybridizations. The other limitation is that it does not make it possible to quantify the proportion of the genome of domestic origin in the admixed individuals. Genotyping of molecular markers theoretically overcomes these limitations and, under certain conditions (sufficient genotyping density and relevant reference populations), allows a fine analysis of the ancestral origins of the individuals studied. The first study of this type carried out in France was based on the genotyping of a panel of 20 SNP markers only, which did not allow a thorough analysis of the genomic composition of the animals studied (Beugin et al., 2017). Our study, like those carried out by Goedbloed, Megens, et al. (2013) and Goedbloed, van Hooft, et al. (2013) and Iacolina et al. (2018) to analyze the introgression in different European WB populations, is based on high‐density molecular genotyping data (several tens of thousands of SNPs), and, as such, represents a major step forward. However, even with such approaches, the detection of introgression remains difficult due to the complex domestication and animal husbandry processes that have taken place in Europe, with significant gene flows between European WBs and European DPs, and between European and Asian DPs (Ai et al., 2013; Chen et al., 2020; Giuffra et al., 2000). In our work, we considered a relatively large number of WBs (362) sampled from different French regions, from different types of management units (WB farms, nature reserves, hunting parks, etc.), and whose chromosome number was known (for 283 out of the 362 individuals studied). In addition, we used genotypes datasets from several DP breeds (both local and commercial) of European as well as of Asian origin to obtain a fairly good representation of the genomic diversity of the *suidae* populations in continental France. This experimental set‐up placed us, a priori, in favorable conditions to study and characterize the different levels of introgression that may have occurred in the recent history of WB populations.

### 4.1 Detection of hybrids using genome‐wide SNP data

Analysis of the WB cluster allowed us to detect 13 outliers (3.6%) without having to arbitrarily set a threshold to qualify certain individuals as outliers. As suggested by the admixture analysis (WB ancestry <58%; Figure 3), these WB_outliers probably had a recent DP ancestry. The 3.6% value is quite close to the one estimated in the past for other populations in Western and Northern Europe: 3.9% on average in Dutch and German populations studied by Goedbloed, van Hooft, et al. (2013); 4% and 6.3% in French and German WB samples studied by Iacolina et al. (2018); in these two studies, however, a threshold was arbitrarily defined to designate hybrid animals). No outlier was detected among the 110 individuals originating from the two wild populations (free‐ranging animals from the WB_Deux‐Sèvres and WB_Ardèche populations). This result suggests that the proportion of recent hybrids would be higher in WB farms. A possible explanation would be that the use of hybridization with DPs is likely to have positive effects in this type of management unit (reduction of inbreeding, use of heterosis as well as the additive effects of genes, which could be important for some traits such as prolificacy or body composition; Frankham, 1995; Iversen et al., 2019). Hybridization could, therefore, be partly voluntary in some WB farms, whereas it would more likely be accidental in the natural environment (escaped pets or DPs). We also found that the main source of recent hybridization was with Asian genotypes (Asian DP ancestry >37% for nine of the 13 outliers). The genomic composition of these nine animals (for the “non WB” part of their genomes) is relatively similar to that of the two so‐called “Vietnamese” animals (part of the genome composed of diverse DP origins and a majority of Meishan; Figure 3). The ease of raising these kinds of animals and their relative resemblance to WBs could explain the use of such genotypes in voluntary hybridizations. However, the analysis of a larger number of animals of this type would be necessary to confirm this hypothesis.

Otherwise, it is noteworthy that: (1) two outliers with 2*n* = 36 chromosomes would not have been detected as “hybrids” (or hybrid offspring) using cytogenetic analyses, and (2) the percentage of outliers (3.6%) was significantly lower than the percentage of “hybrids” estimated using cytogenetic analyses (28% of WBs with 2*n* = 37 or 38 chromosomes in our sample of genotyped individuals). This difference could be explained by the fact that the majority of WB_38 and WB_37 were the result of ancient hybridization events followed by many generations of backcross with WB, which is consistent with the observation that some of the WB_38 or WB_37 individuals were even less admixed than some WBs with 2*n* = 36 chromosomes (Figure 2). Another explanation would be that WB_37 and WB_38 individuals with very low DP admixture levels are in fact the products of hybridizations with WBs from Central and Eastern European regions (which often present a 2*n* = 38 karyotype; however, our results do not support this hypothesis: see Section 4.4).

The 13 outliers should not be considered as WBs and should not be used in WB farms, even those with 2*n* = 36 chromosomes. Overall, our results show that detecting WB × DP hybridization using cytogenetic techniques only is neither accurate nor reliable enough and that an evolution of the detection techniques should be considered.

### 4.2 Adaptive introgression in WBs

Our analyses revealed multiple regions of domestic ancestry in WBs, with the strongest introgression signal observed on chromosome 13. This genomic region of 5.6 Mb (from 83.5 to 89.1 Mb) overlaps with a previously reported QTL for body weight at birth (86.0 to 94.2 Mb; Yue et al., 2003). This region is also adjacent to other QTLs for the number of piglets born alive (NBA: 89.4 to 89.7 Mb, Onteru et al., 2012 and 72.0 to 82.6 Mb, Ma et al., 2018), uterine horn length (80.0 to 80.2 Mb; Rosendo et al., 2012), and age at puberty (92.5 to 92.8 Mb; Bidanel et al., 2008). The introgression of favorable alleles of domestic pig origin(s) into WBs could have induced an improvement in their fitness, which may explain the demographic growth of populations observed in recent decades (Albrycht et al., 2016; Massei et al., 2015). If introgressed alleles are not counter‐selected, they will become increasingly common in WB populations, raising important questions about their future management.

The most frequently introgressed region of 5.6 Mb comprises 18 genes. One of these genes (PLSCR4, involved in the uterine function) could be the target of positive selection. In rat uterus, PLSCR4 provides a dynamic mechanism by which aminophospholipid translocation can be regulated, thereby modulating the activity of various membrane proteins that are involved in inflammation and coagulation events in the uterus (Phillippe et al., 2006). Three other genes adjacent to the 5.6 Mb region, that is, RBP1 (80.4 Mb), RBP2 (80.3 Mb) (functionally related to uterus development), and CLSTN2 (80.8 to 81.5 Mb) are mainly expressed in pig ovary (Li et al., 2017) and could also be associated with the reproductive traits mentioned above (including NBA).

### 4.3 Variation of the level of admixture between different wild boar populations

Among the 349 WBs studied (WB_Cluster), 210 (60%) showed traces of introgression. Conversely, 139 (40%) could be considered as unadmixed WBs (i.e., animals for which the 95% confidence intervals for the Admixture Q scores overlapped 0.99). Even if the percentage of admixed WBs was relatively large in our sample, the proportion of genomes of DP ancestry was quite low (around 6% on average). One explanation would be that DP × WB hybridizations regularly occurred throughout the history of the different populations, but with moderate intensity, and were followed by many generations of backcrosses, contributing to the reduction, generation after generation, of the proportion of the genome of DP origin in the offspring. Another possibility would be the counter‐selection of domestic traits in wild populations. However, we did not observe any genomic region free of DP introgression, which does not support the latter hypothesis. Otherwise, the percentage of admixed individuals (60%) must be considered with caution since our sample was not designed to be strictly representative of French WB populations, but, instead, to generate favorable conditions to detect and characterize DP × WB hybridization. This explains why the proportion of individuals with 2*n* = 37 or 38 chromosomes was significantly higher in our sample of genotyped animals (28%, *n* = 283) than in the sample of animals having undergone cytogenetic evaluation between 2008 and 2020 (15.3%; *n* = 4894).

Fifty‐six WB were sampled in seven management units considered to be preserved from hybridization based on 12 years of cytogenetic controls (Table 1). The low proportion of the genome of DP origin (2% on average vs. 6% globally; Figure S7) and the absence of outliers in this sample is probably the result of good management practices carried out in these units in order to preserve the genetic integrity of the wild species.

The case of the WB_Deux‐Sèvres population (nature reserve in western France) is interesting. The first cytogenetic survey carried out in 1989–1990 in this population had not detected any hybrids (WBs with 2*n* = 37 or 38 chromosomes; Darre et al., 1992). During the recent period (2008–2020), the proportion of “cytogenetical hybrids” (or hybrid offspring) was quite high (40%), whereas the proportion of the genome of DP origin in these animals was very low (1% on average). This population originates from a state‐owned forest protected from silvicultural activities and classified as a national hunting and wildlife reserve since 1973. The “Lothar” and “Martin” storms that hit France in December 1999 (called “the storms of the century”) (Salomon, 2000; Ulbrich et al., 2001), which were particularly severe in that area, may explain the rapid increase in the number of hybrids (2*n* = 37) at the beginning of the 2000s. Indeed, the destruction of fences induced by these devastating storms may have facilitated interactions with domestic and/or pet pigs. These accidental hybridizations would have been followed over the next 20 years by many generations of backcrossing, contributing to a rapid and strong dilution of the proportion of the genome of DP origin in this population.

Analyses of animals from the wild population of Ardèche (unprotected area in southeast France) revealed a proportion of the genome of DP origin (7% on average) that was significantly higher than in the other wild population of Deux‐Sèvres (Figure S7b). None of the animals in this population was classified as “unadmixed” (indicating that traces of admixture were detected in all of the 82 genotyped WBs). These results suggest that hybridizations with DPs were more frequent in this area than in others, which could possibly explain, in part, the considerable demographic growth of this WB population. However, admixture may not always be beneficial. It has been observed, for example, that domestic introgression may lead to increased wildlife susceptibility to infectious diseases (introgression of susceptibility genes, which may modify the immune response of admixed individuals; Goedbloed et al., 2015). This may have been one reason (among others, including direct contacts between DPs and WBs) for the appearance of cases of edema disease in 2013 in this Ardèche population (Decors et al., 2015). Edema disease is relatively common in DP (Luppi et al., 2016) but had never been diagnosed in WB before that date. Comparison of these WB_Ardèche samples (population that was not subject to any particular management procedure) with those of WB_Deux‐Sèvres or WB_Preserved (in which significant efforts have been made to preserve natural populations for a long time) suggests that the lack of rigorous management procedures represents a threat to the genetic integrity of WB populations.

### 4.4 Origin of 2*n* = 37 or 38 WB

The geographical distribution and genetic diversity of WB populations across Europe were mainly shaped during the last glacial periods. According to different studies, current European WB populations originate from refuges in three southern European regions: the Iberian Peninsula, Italy, and the Balkans (Veličković et al., 2016). Different karyotypes have been described in European WBs, with variable frequencies depending on the origin of the populations. The frequency of 2*n* = 38 animals appears to be quite high in some Central and Eastern European populations (Aravena & Skewes, 2007), whereas this frequency was very low or null in Western European populations (especially in French populations). We can, therefore, hypothesize that some individuals with 37 or 38 chromosomes in French WB populations may be the products of hybridization(s) with WBs originating from Central or Eastern European regions (such animals could have been imported into France, e.g., to supply some hunting parks). To elucidate this point, we analyzed the ancestral origin of French WBs along chromosomes 15 and 17. We paid particular attention to the ancestral origin of the pericentromic regions of these chromosomes, which, due to a low recombination rate (Mary et al., 2014), should retain their ancestral haplotypes. Our analyses revealed very different results depending on the number of chromosomes present in the individuals studied (Figure S8). The ancestral origin of pericentromeric markers is exclusively “wild” for WB_36, and we observed much lower proportions of wild ancestry on that particular region for WB_37 and WB_38 animals. For WB_38, there is a discrepancy between the expected and observed proportions of the genome of “domestic” origin in the pericentromeric regions of chromosomes 15 and 17 (100% vs. 67.1%, respectively). As previously mentioned, one explanation could be that some of the WB_38 (and possibly some WB_37) individuals were the result of hybridization(s) with Central or Eastern European boars with 2*n* = 38 chromosomes. Unfortunately, our data do not allow to test this hypothesis. Another explanation could be that the hybridization events at the origin of some WB_37 or WB_38 animals are too old and that the genotyping density we used was insufficient to detect “domestic” haplotypes at these particular genomic regions. A final explanation could be the nature of the “wild” reference population used with ELAI (animals considered “unadmixed” after Admixture analyses, including animals with 37 and 38 chromosomes).

To avoid this potential source of bias and the very long computation times required by ELAI software, we used the LAMP algorithm with WB_36 individuals only as the reference population. The in silico prediction of the number of chromosomes using LAMP was very accurate for WB_36 and WB_38 animals (Table 2). The error rate was higher for WB_37, but these animals were much less frequent in the populations analyzed than the WB_36. This explains why, overall, the CR was quite high (95%). Local ancestry analyses carried out using LAMP confirmed that the pericentromeric regions of chromosomes 15 and 17 in most WBs with 2*n* = 37 or 38 chromosomes were of domestic origin. We also observed that the WB ancestry is the highest for WB_36 and that it decreases when the number of chromosomes increases (Figure S7a).

Overall, our analyses suggest that the normal (ancestral) diploid number of chromosomes in French WBs is 2*n* = 36 and that the higher number of chromosomes observed in some individuals (WB_37 and WB_38) is most probably the result of past (mostly ancient) hybridization events with DP. In future, LAMP could be used to routinely predict the number of chromosomes in individuals analyzed by genotyping as part of future programs aimed at monitoring the genetic integrity of WB populations.

## 5 CONCLUSION

In conclusion, the discrepancies observed in our study between the results of cytogenetic evaluations and analyses based on genome‐wide molecular genotyping, the additional information provided by SNP genotyping (determination of the composition of the animal genome, opportunity to differentiate individuals originating from recent or older hybridization events, etc.), and the possibility offered by DNA‐based techniques to work from skin biopsies or other biological samples taken from dead animals, argue in favor of an evolution of the methods used in WB population monitoring programs in France and in other European countries. Indeed, we have demonstrated that karyotyping does not detect some highly admixed individuals with 2*n* = 36 chromosomes. This is very unlikely with genotyping. The only (relatively minor) problem with genotyping‐based monitoring approaches would be the release of relatively few unadmixed but 37‐chromosome individuals for breeding. For example, among the 139 individuals considered as unadmixed based on the admixture analysis, five were considered in silico as having 36 chromosomes, while those individuals were cytogenetically diagnosed with 2*n* = 37 chromosomes. In other words, our results suggest that while the use of genotyping data would probably be effective in preserving the genetic integrity of WB populations, it would probably not make it possible to fully preserve the original chromosomal status of the *Sus scrofa* species (2*n* = 36). However, with an average 15.3% proportion of animals with 2*n* = 37 or 38 chromosomes (and considering the substantial year‐to‐year variation of this proportion and the fact that it can be as high as 40% in some management units), the cytogenetic integrity of French WB populations already seems relatively compromised.

The technical approach used in our study was the same as that used on a large scale for the genomic selection of commercial swine populations. As compared to other molecular approaches (e.g., multiplex STR‐typing and real‐time PCR evaluated by Lorenzini et al., 2020), which would probably be more affordable in the short term, genome‐wide genotyping can substantially improve the precision with which the spatio‐temporal levels of hybridization are quantified. This could also provide the opportunity to carry out a selection against the DP haplotypes that could potentially increase WB fitness. The cost of these technologies remains significant but has steadily declined in recent years. Their deployment for the control of WB populations could, therefore, be reasonably envisaged in the relatively short term. This would improve the study and management of natural and farmed WB populations, provide a better understanding of the nature and dynamics of interactions between farmed and wild populations of WB, as well as the evolutionary consequences of hybridization, and possibly meet some of the needs for forensic expertise (Lorenzini et al., 2020).

## CONFLICT OF INTEREST

The authors certify that they have no affiliations with or involvement in any organization or entity with any financial interest or non‐financial interest in the subject matter or materials discussed in this manuscript.

## Supporting information


Figure S1

Figure S2

Figure S3

Figure S4

Figure S5

Figure S6

Figure S7

Figure S8

Table S1

Table S2

## Data Availability

The complete genotypes dataset produced in this paper is described in a data paper (Iannuccelli et al., 2022).

## References

[eva13432-bib-0001] Ai, H. , Huang, L. , & Ren, J. (2013). Genetic diversity, linkage disequilibrium and selection signatures in Chinese and Western pigs revealed by genome‐wide SNP markers. PLoS One, 8(2), e56001. 10.1371/journal.pone.0056001 23409110 PMC3567019

[eva13432-bib-0002] Albrycht, M. , Merta, D. , Bobek, J. , & Ulejczyk, S. (2016). The demographic pattern of wild boars (*Sus scrofa*) inhabiting fragmented forest in North‐Eastern Poland. Baltic Forestry, 22(2), 251–258.

[eva13432-bib-0003] Alexander, D. H. , Novembre, J. , & Lange, K. (2009). Fast model‐based estimation of ancestry in unrelated individuals. Genome Research, 19(9), 1655–1664. 10.1101/gr.094052.109 19648217 PMC2752134

[eva13432-bib-0004] Aravena, P. , & Skewes, O. (2007). European wild boar purebred and *Sus scrofa* intercrosses. Discrimination proposals. A review. Agro Ciencia, 23, 133–147.

[eva13432-bib-0005] Barrios‐Garcia, M. N. , & Ballari, S. A. (2012). Impact of wild boar (*Sus scrofa*) in its introduced and native range: A review. Biological Invasions, 14(11), 2283–2300. 10.1007/s10530-012-0229-6

[eva13432-bib-0006] Behr, A. A. , Liu, K. Z. , Liu‐Fang, G. , Nakka, P. , & Ramachandran, S. (2016). pong: Fast analysis and visualization of latent clusters in population genetic data. Bioinformatics, 32(18), 2817–2823. 10.1093/bioinformatics/btw327 27283948 PMC5018373

[eva13432-bib-0007] Beugin, M.‐P. , Baubet, E. , Dufaure De Citres, C. , Kaerle, C. , Muselet, L. , Klein, F. , & Queney, G. (2017). A set of 20 multiplexed single nucleotide polymorphism (SNP) markers specifically selected for the identification of the wild boar (*Sus scrofa scrofa*) and the domestic pig (*Sus scrofa domesticus*). Conservation Genetics Resources, 9(4), 671–675. 10.1007/s12686-017-0738-9

[eva13432-bib-0008] Bidanel, J. P. , Rosendo, A. , Iannuccelli, N. , Riquet, J. , Gilbert, H. , Caritez, J. C. , Billon, Y. , Amigues, Y. , Prunier, A. , & Milan, D. (2008). Detection of quantitative trait loci for teat number and female reproductive traits in Meishan × Large White F2 pigs. Animal, 2(6), 813–820. 10.1017/S1751731108002097 22443659

[eva13432-bib-0009] Blome, S. , Franzke, K. , & Beer, M. (2020). African swine fever—A review of current knowledge. Virus Research, 287, 198099. 10.1016/j.virusres.2020.198099 32755631

[eva13432-bib-0010] Charlez, A. (2010). L'élevage et la commercialisation des sangliers. Faune Sauvage, 288(288), 48–55.

[eva13432-bib-0011] Chen, H. , Huang, M. , Yang, B. , Wu, Z. , Deng, Z. , Hou, Y. , Ren, J. , & Huang, L. (2020). Introgression of Eastern Chinese and Southern Chinese haplotypes contributes to the improvement of fertility and immunity in European modern pigs. GigaScience, 9(3), giaa014. 10.1093/gigascience/giaa014 32141510 PMC7059266

[eva13432-bib-0012] Darre, R. , Berland, H. M. , & Goustat, P. (1992). Statut chromosomique des populations de sangliers sauvages et d'élevages en France. Revue de Médecine Vétérinaire, 3, 225–232.

[eva13432-bib-0013] Decors, A. , Richomme, C. , Morvan, H. , Botteron, C. , Nicolier, A. , Rambaud, F. , Berny, P. , Gault, G. , Belli, P. , Le Potier, M.‐F. , Fach, P. , Delannoy, S. , Baubet, E. , Etienne, F. , & Lemberger, K. (2015). Diagnosing a health problem in wildlife: Example of edema disease in wild boar (*Sus scrofa*) in Ardèche, France. Bulletin Épidémiologique, Santé Animale et Alimentation Anses‐DGAl, 69, 2–7.

[eva13432-bib-0014] Delibes‐Mateo, M. , & Delibes, A. (2013). Pets becoming established in the wild: Free–living Vietnamese potbellied pigs in Spain. Animal Biodiversity and Conservation, 36, 209–215.

[eva13432-bib-0015] Ducos, A. , Revay, T. , Kovacs, A. , Hidas, A. , Pinton, A. , Bonnet‐Garnier, A. , Molteni, L. , Slota, E. , Switonski, M. , Arruga, M. V. , van Haeringen, W. A. , Nicolae, I. , Chaves, R. , Guedes‐Pinto, H. , Andersson, M. , & Iannuzzi, L. (2008). Cytogenetic screening of livestock populations in Europe: An overview. Cytogenetic and Genome Research, 120(1–2), 26–41. 10.1159/000118738 18467823

[eva13432-bib-0016] Francis, R. M. (2017). pophelper: An R package and web app to analyse and visualize population structure. Molecular Ecology Resources, 17(1), 27–32. 10.1111/1755-0998.12509 26850166

[eva13432-bib-0017] Frankham, R. (1995). Conservation genetics. Annual Review of Genetics, 29(1), 305–327. 10.1146/annurev.ge.29.120195.001513 8825477

[eva13432-bib-0018] Frantz, L. A. F. , Schraiber, J. G. , Madsen, O. , Megens, H.‐J. , Cagan, A. , Bosse, M. , Paudel, Y. , Crooijmans, R. P. M. A. , Larson, G. , & Groenen, M. A. M. (2015). Evidence of long‐term gene flow and selection during domestication from analyses of Eurasian wild and domestic pig genomes. Nature Genetics, 47(10), 1141–1148. 10.1038/ng.3394 26323058

[eva13432-bib-0019] Frantz, L. A. F. , Haile, J. , Lin, A. T. , Scheu, A. , Geörg, C. , Benecke, N. , Alexander, M. , Linderholm, A. , Mullin, V. E. , Daly, K. G. , Battista, V. M. , Price, M. , Gron, K. J. , Alexandri, P. , Arbogast, R.‐M. , Arbuckle, B. , Bӑlӑşescu, A. , Barnett, R. , Bartosiewicz, L. , … Larson, G. (2019). Ancient pigs reveal a near‐complete genomic turnover following their introduction to Europe. Proceedings of the National Academy of Sciences, 116(35), 17231–17238. 10.1073/pnas.1901169116 PMC6717267 31405970

[eva13432-bib-0021] Gillespie, A. V. , Grove‐White, D. H. , & Williams, H. J. (2015). Husbandry, health and biosecurity of the smallholder and pet pig population in England. The Veterinary Record, 177(2), 47. 10.1136/vr.102759 26116269

[eva13432-bib-0022] Giuffra, E. , Kijas, J. M. H. , Amarger, V. , Carlborg, Ö. , Jeon, J.‐T. , & Andersson, L. (2000). The origin of the domestic pig: Independent domestication and subsequent introgression. Genetics, 154(4), 1785–1791. 10747069 10.1093/genetics/154.4.1785 PMC1461048

[eva13432-bib-0023] Goedbloed, D. J. , Megens, H. J. , Van Hooft, P. , Herrero‐Medrano, J. M. , Lutz, W. , Alexandri, P. , Crooijmans, R. P. M. A. , Groenen, M. , Van Wieren, S. E. , Ydenberg, R. C. , & Prins, H. H. T. (2013). Genome‐wide single nucleotide polymorphism analysis reveals recent genetic introgression from domestic pigs into Northwest European wild boar populations. Molecular Ecology, 22(3), 856–866. 10.1111/j.1365-294X.2012.05670.x 22731769

[eva13432-bib-0024] Goedbloed, D. J. , van Hooft, P. , Megens, H.‐J. , Langenbeck, K. , Lutz, W. , Crooijmans, R. P. , van Wieren, S. E. , Ydenberg, R. C. , & Prins, H. H. (2013). Reintroductions and genetic introgression from domestic pigs have shaped the genetic population structure of Northwest European wild boar. BMC Genetics, 14(1), 43. 23688182 10.1186/1471-2156-14-43 PMC3663677

[eva13432-bib-0025] Goedbloed, D. J. , van Hooft, P. , Lutz, W. , Megens, H.‐J. , van Wieren, S. E. , Ydenberg, R. C. , & Prins, H. H. T. (2015). Increased Mycoplasma hyopneumoniae disease prevalence in domestic hybrids among free‐living wild boar. EcoHealth, 12(4), 571–579. 10.1007/s10393-015-1062-z 26391376

[eva13432-bib-0026] Guan, Y. (2014). Detecting structure of haplotypes and local ancestry. Genetics, 196(3), 625–642. 10.1534/genetics.113.160697 24388880 PMC3948796

[eva13432-bib-0027] Gustavsson, I. (1988). Standard karyotype of the domestic pig. Hereditas, 109(2), 151–157. 3230021 10.1111/j.1601-5223.1988.tb00351.x

[eva13432-bib-0028] Hars, J. , & Rossi, S. (2010). Évaluation des risques sanitaires liés à l'augmentation des effectifs de sangliers en France. Faune Sauvage, 288, 23–28.

[eva13432-bib-0029] Iacolina, L. , Pertoldi, C. , Amills, M. , Kusza, S. , Megens, H.‐J. , Bâlteanu, V. A. , Bakan, J. , Cubric‐Curic, V. , Oja, R. , Saarma, U. , Scandura, M. , Šprem, N. , & Stronen, A. V. (2018). Hotspots of recent hybridization between pigs and wild boars in Europe. Scientific Reports, 8(1), 17372. 10.1038/s41598-018-35865-8 30478374 PMC6255867

[eva13432-bib-0030] Iannuccelli, N. , Mary, N. , Bonnet, N. , Petit, G. , Valle, C. , Ducos, A. , & Riquet, J. (2022). Genotyping data of French wild boar populations using porcine genome‐wide genotyping array. BMC Research Notes, 15(1), 170. 10.1186/s13104-022-06052-w 35562745 PMC9102940

[eva13432-bib-0031] Iversen, M. W. , Nordbø, Ø. , Gjerlaug‐Enger, E. , Grindflek, E. , Lopes, M. S. , & Meuwissen, T. (2019). Effects of heterozygosity on performance of purebred and crossbred pigs. Genetics, Selection, Evolution, 51(1), 8. 10.1186/s12711-019-0450-1 PMC6396501 30819106

[eva13432-bib-0032] Khederzadeh, S. , Kusza, S. , Huang, C. , Markov, N. , Scandura, M. , Babaev, E. , Šprem, N. , Seryodkin, I. V. , Paule, L. , Esmailizadeh, A. , Xie, H. , & Zhang, Y. (2019). Maternal genomic variability of the wild boar (*Sus scrofa*) reveals the uniqueness of East‐Caucasian and Central Italian populations. Ecology and Evolution, 9(17), 9467–9478. 10.1002/ece3.5415 31534669 PMC6745674

[eva13432-bib-0033] Lee, S. H. , Seo, D. W. , Cho, E. S. , Choi, B. H. , Kim, Y. M. , Hong, J. K. , Han, H. D. , Jung, Y. B. , Kim, D. J. , Choi, T. J. , & Lee, S. H. (2020). Genetic diversity and ancestral study for Korean Native Pigs Using 60K SNP chip. Animals, 10(5), 760. 10.3390/ani10050760 PMC7277343 32349346

[eva13432-bib-0034] Li, M. , Chen, L. , Tian, S. , Lin, Y. , Tang, Q. , Zhou, X. , Li, D. , Yeung, C. K. L. , Che, T. , Jin, L. , Fu, Y. , Ma, J. , Wang, X. , Jiang, A. , Lan, J. , Pan, Q. , Liu, Y. , Luo, Z. , Guo, Z. , … Li, X. (2017). Comprehensive variation discovery and recovery of missing sequence in the pig genome using multiple de novo assemblies. Genome Research, 27(5), 865–874. 10.1101/gr.207456.116 27646534 PMC5411780

[eva13432-bib-0035] Lorenzini, R. , Fanelli, R. , Tancredi, F. , Siclari, A. , & Garofalo, L. (2020). Matching STR and SNP genotyping to discriminate between wild boar, domestic pigs and their recent hybrids for forensic purposes. Scientific Reports, 10(1), 3188. 10.1038/s41598-020-59644-6 32081854 PMC7035276

[eva13432-bib-0036] Luppi, A. , Gibellini, M. , Gin, T. , Vangroenweghe, F. , Vandenbroucke, V. , Bauerfeind, R. , Bonilauri, P. , Labarque, G. , & Hidalgo, Á. (2016). Prevalence of virulence factors in enterotoxigenic *Escherichia coli* isolated from pigs with post‐weaning diarrhoea in Europe. Porcine Health Management, 2, 20. 10.1186/s40813-016-0039-9 28405446 PMC5382528

[eva13432-bib-0037] Ma, X. , Li, P. H. , Zhu, M. X. , He, L. C. , Sui, S. P. , Gao, S. , Su, G. S. , Ding, N. S. , Huang, Y. , Lu, Z. Q. , Huang, X. G. , & Huang, R. H. (2018). Genome‐wide association analysis reveals genomic regions on chromosome 13 affecting litter size and candidate genes for uterine horn length in Erhualian pigs. Animal, 12(12), 2453–2461. 10.1017/S1751731118000332 29534777

[eva13432-bib-0038] Mary, N. , Barasc, H. , Ferchaud, S. , Billon, Y. , Meslier, F. , Robelin, D. , Calgaro, A. , Loustau‐Dudez, A.‐M. , Bonnet, N. , Yerle, M. , Acloque, H. , Ducos, A. , & Pinton, A. (2014). Meiotic recombination analyses of individual chromosomes in male domestic pigs (*Sus scrofa domestica*). PLoS One, 9(6), e99123. 10.1371/journal.pone.0099123 24919066 PMC4053413

[eva13432-bib-0039] Massei, G. , Kindberg, J. , Licoppe, A. , Gačić, D. , Šprem, N. , Kamler, J. , Baubet, E. , Hohmann, U. , Monaco, A. , Ozoliņš, J. , Cellina, S. , Podgórski, T. , Fonseca, C. , Markov, N. , Pokorny, B. , Rosell, C. , & Náhlik, A. (2015). Wild boar populations up, numbers of hunters down? A review of trends and implications for Europe: Wild boar and hunter trends in Europe. Pest Management Science, 71(4), 492–500. 10.1002/ps.3965 25512181

[eva13432-bib-0040] Mercat, M.‐J. , Labrune, Y. , Feve, K. , Ferchaud, S. , Lenoir, H. , & Riquet, J. (2020). Caractérisation génomique des races locales porcines et de leurs semences stockées dans la Cryobanque Nationale. Journées de la Recherche Porcine, 38, 217–223.

[eva13432-bib-0041] Muñoz, M. , Bozzi, R. , García‐Casco, J. , Núñez, Y. , Ribani, A. , Franci, O. , García, F. , Škrlep, M. , Schiavo, G. , Bovo, S. , Utzeri, V. J. , Charneca, R. , Martins, J. M. , Quintanilla, R. , Tibau, J. , Margeta, V. , Djurkin‐Kušec, I. , Mercat, M. J. , Riquet, J. , … Óvilo, C. (2019). Genomic diversity, linkage disequilibrium and selection signatures in European local pig breeds assessed with a high density SNP chip. Scientific Reports, 9(1), 13546. 10.1038/s41598-019-49830-6 31537860 PMC6753209

[eva13432-bib-0042] Nombela, J. A. , Murcia, C. R. , Abaigar, T. , & Vericad, J. (1990). Cytogenetic analysis (GTG, CBG and NOR bands) of a wild boar population (*Sus scrofa scrofa*) with chromosomal polymorphism in the south‐east of Spain. Genetics Selection Evolution, 22, 1–9.

[eva13432-bib-0043] Onteru, S. K. , Fan, B. , Du, Z.‐Q. , Garrick, D. J. , Stalder, K. J. , & Rothschild, M. F. (2012). A whole‐genome association study for pig reproductive traits: WGAS for pig reproductive traits. Animal Genetics, 43(1), 18–26. 10.1111/j.1365-2052.2011.02213.x 22221021

[eva13432-bib-0044] Østevik, L. , Elmas, C. , & Rubio‐Martinez, L. M. (2012). Castration of the Vietnamese pot‐bellied boar: 8 cases. The Canadian Veterinary Journal, 53(9), 943–948. 23450857 PMC3418779

[eva13432-bib-0045] Petit, G. , Grosbois, V. , Chalvet‐Monfray, K. , Ducos, A. , Desmecht, D. , Martineau, G.‐P. , & Decors, A. (2020). Polymorphism of the alpha‐1‐fucosyltransferase (FUT1) gene in several wild boar (*Sus scrofa*) populations in France and link to edema disease. Research in Veterinary Science, 131, 78–86. 10.1016/j.rvsc.2020.03.025 32311589

[eva13432-bib-0046] Phillippe, M. , Bradley, D. F. , Ji, H. , Oppenheimer, K. H. , & Chien, E. K. (2006). Phospholipid scramblase isoform expression in pregnant rat uterus. Journal of the Society for Gynecologic Investigation, 13(7), 497–501. 10.1016/j.jsgi.2006.06.002 16979355

[eva13432-bib-0047] Root, T. L. , Price, J. T. , Hall, K. R. , Schneider, S. H. , Rosenzweig, C. , & Pounds, J. A. (2003). Fingerprints of global warming on wild animals and plants. Nature, 421(6918), 57–60. 10.1038/nature01333 12511952

[eva13432-bib-0048] Rosendo, A. , Iannuccelli, N. , Gilbert, H. , Riquet, J. , Billon, Y. , Amigues, Y. , Milan, D. , & Bidanel, J. P. (2012). Microsatellite mapping of quantitative trait loci affecting female reproductive tract characteristics in Meishan × Large White F(2) pigs. Journal of Animal Science, 90(1), 37–44. 10.2527/jas.2011-3989 21948608

[eva13432-bib-0049] Salomon, J.‐N. (2000). La “tempête du siècle” (27 et 28 décembre 1999) et notamment en Aquitaine. Travaux du Laboratoire de Géographie Physique Appliquée, 19(1), 31–53. 10.3406/tlgpa.2000.975

[eva13432-bib-0050] Sankararaman, S. , Sridhar, S. , Kimmel, G. , & Halperin, E. (2008). Estimating local ancestry in admixed populations. The American Journal of Human Genetics, 82(2), 290–303. 10.1016/j.ajhg.2007.09.022 18252211 PMC2664993

[eva13432-bib-0051] Scrucca, L. , Fop, M. , Murphy, T. B. , & Raftery, A. E. (2016). mclust 5: Clustering, classification and density estimation using gaussian finite mixture models. The R Journal, 8(1), 289. 10.32614/RJ-2016-021 27818791 PMC5096736

[eva13432-bib-0052] Tynes, V. V. (2001). Behavior of miniature pet pigs. Veterinary Clinics of North America: Exotic Animal Practice, 4(3), 713–734. 10.1016/S1094-9194(17)30033-6 11601110

[eva13432-bib-0053] Ulbrich, U. , Fink, A. H. , Klawa, M. , & Pinto, J. G. (2001). Three extreme storms over Europe in December 1999. Weather, 56(3), 70–80. 10.1002/j.1477-8696.2001.tb06540.x

[eva13432-bib-0054] Veličković, N. , Ferreira, E. , Djan, M. , Ernst, M. , Obreht Vidaković, D. , Monaco, A. , & Fonseca, C. (2016). Demographic history, current expansion and future management challenges of wild boar populations in the Balkans and Europe. Heredity, 117(5), 348–357. 10.1038/hdy.2016.53 27436523 PMC5061920

[eva13432-bib-0055] Wayne, R. K. , & Shaffer, H. B. (2016). Hybridization and endangered species protection in the molecular era. Molecular Ecology, 25(11), 2680–2689. 10.1111/mec.13642 27064931

[eva13432-bib-0056] Yamamoto, D. (2017). Wild boar. Reaktion Books.

[eva13432-bib-0057] Yue, G. H. , Russo, V. , Davoli, R. , Sternstein, I. , Brunsch, C. , Schröffelová, D. , Stratil, A. , Moser, G. , Bartenschlager, H. , Reiner, G. , & Geldermann, H. (2003). Linkage and QTL mapping for Sus scrofa chromosome 13. Journal of Animal Breeding and Genetics, 120, 103–110. 10.1046/j.0931-2668.2003.00430.x

[eva13432-bib-0058] Zheng, X. , Levine, D. , Shen, J. , Gogarten, S. M. , Laurie, C. , & Weir, B. S. (2012). A high‐performance computing toolset for relatedness and principal component analysis of SNP data. Bioinformatics, 28(24), 3326–3328. 10.1093/bioinformatics/bts606 23060615 PMC3519454

