# Supplementary material for: Genome‐wide analysis of hybridization in wild boar populations reveals adaptive introgression from domestic pig
Source: Evol Appl. 2022 Jul 2;15(7):1115–28. doi: 10.1111/eva.13432 (PMC9309462; doi:10.1111/eva.13432)
Supplement: Supplementary file 1 — Figure S1 [file EVA-15-1115-s006.pptx]

## Slide 1
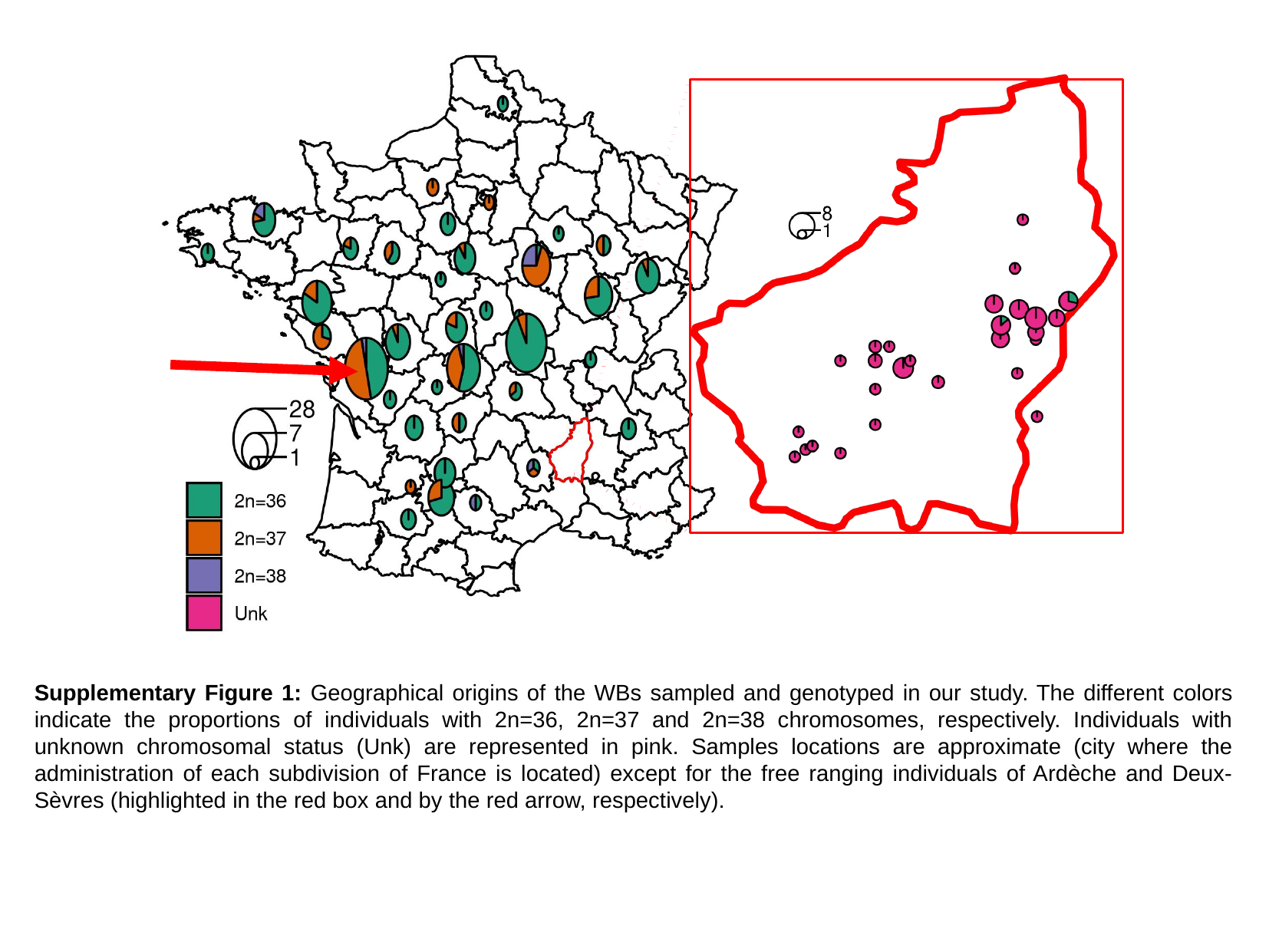

Supplementary Figure 1: Geographical origins of the WBs sampled and genotyped in our study. The different colors indicate the proportions of individuals with 2n=36, 2n=37 and 2n=38 chromosomes, respectively. Individuals with unknown chromosomal status (Unk) are represented in pink. Samples locations are approximate (city where the administration of each subdivision of France is located) except for the free ranging individuals of Ardèche and Deux-Sèvres (highlighted in the red box and by the red arrow, respectively).
