# Supplementary material for: Genome‐wide analysis of hybridization in wild boar populations reveals adaptive introgression from domestic pig
Source: Evol Appl. 2022 Jul 2;15(7):1115–28. doi: 10.1111/eva.13432 (PMC9309462; doi:10.1111/eva.13432)
Supplement: Supplementary file 2 — Figure S2 [file EVA-15-1115-s008.pptx]

## Slide 1
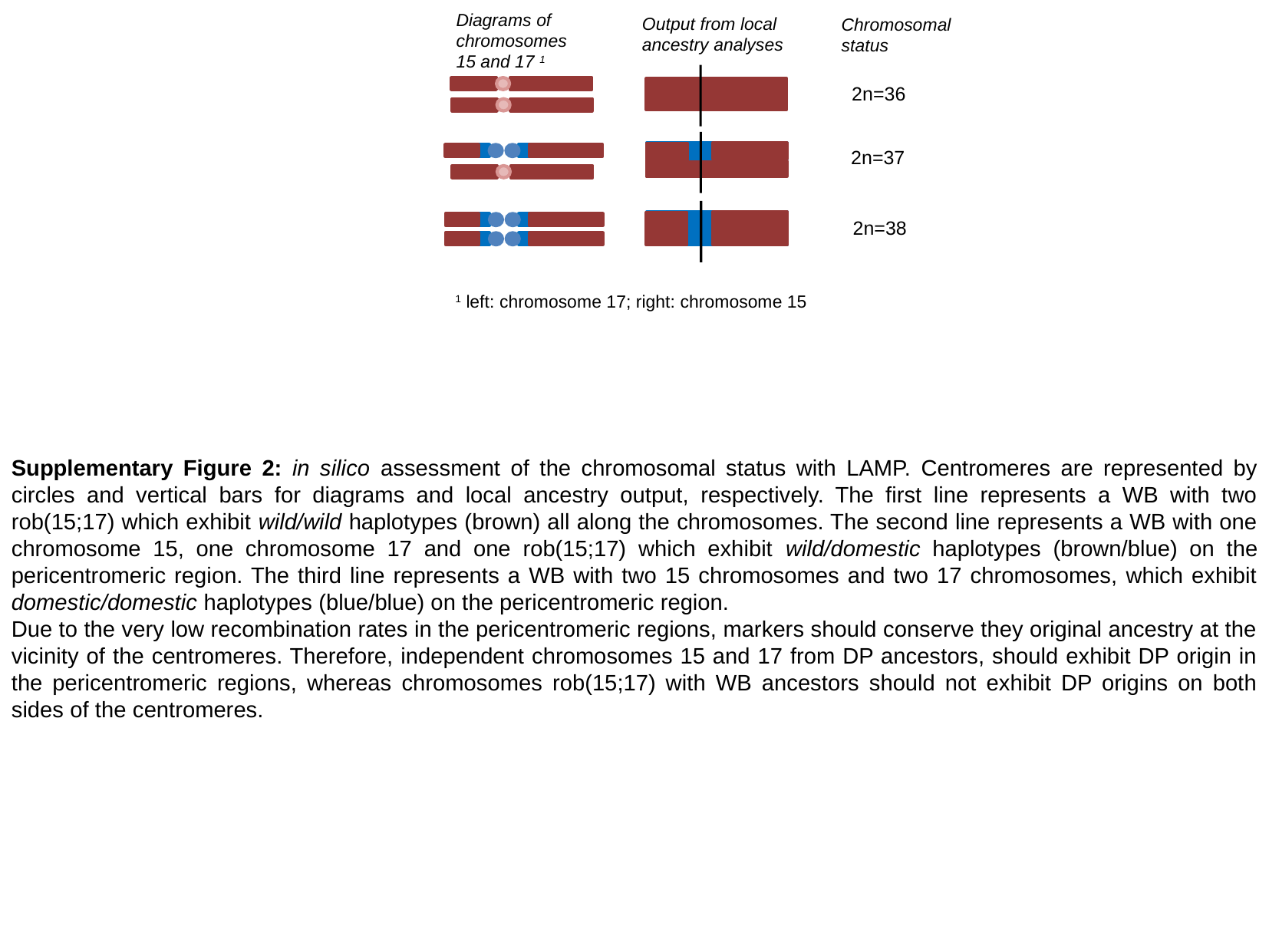

Diagrams of chromosomes 15 and 17 1
Output from local ancestry analyses
Chromosomal status
2n=36
2n=37
2n=38
1 left: chromosome 17; right: chromosome 15
Supplementary Figure 2: in silico assessment of the chromosomal status with LAMP. Centromeres are represented by circles and vertical bars for diagrams and local ancestry output, respectively. The first line represents a WB with two rob(15;17) which exhibit wild/wild haplotypes (brown) all along the chromosomes. The second line represents a WB with one chromosome 15, one chromosome 17 and one rob(15;17) which exhibit wild/domestic haplotypes (brown/blue) on the pericentromeric region. The third line represents a WB with two 15 chromosomes and two 17 chromosomes, which exhibit domestic/domestic haplotypes (blue/blue) on the pericentromeric region.
Due to the very low recombination rates in the pericentromeric regions, markers should conserve they original ancestry at the vicinity of the centromeres. Therefore, independent chromosomes 15 and 17 from DP ancestors, should exhibit DP origin in the pericentromeric regions, whereas chromosomes rob(15;17) with WB ancestors should not exhibit DP origins on both sides of the centromeres.
