# Supplementary material for: Genome‐wide analysis of hybridization in wild boar populations reveals adaptive introgression from domestic pig
Source: Evol Appl. 2022 Jul 2;15(7):1115–28. doi: 10.1111/eva.13432 (PMC9309462; doi:10.1111/eva.13432)
Supplement: Supplementary file 3 — Figure S3 [file EVA-15-1115-s002.pptx]

## Slide 1
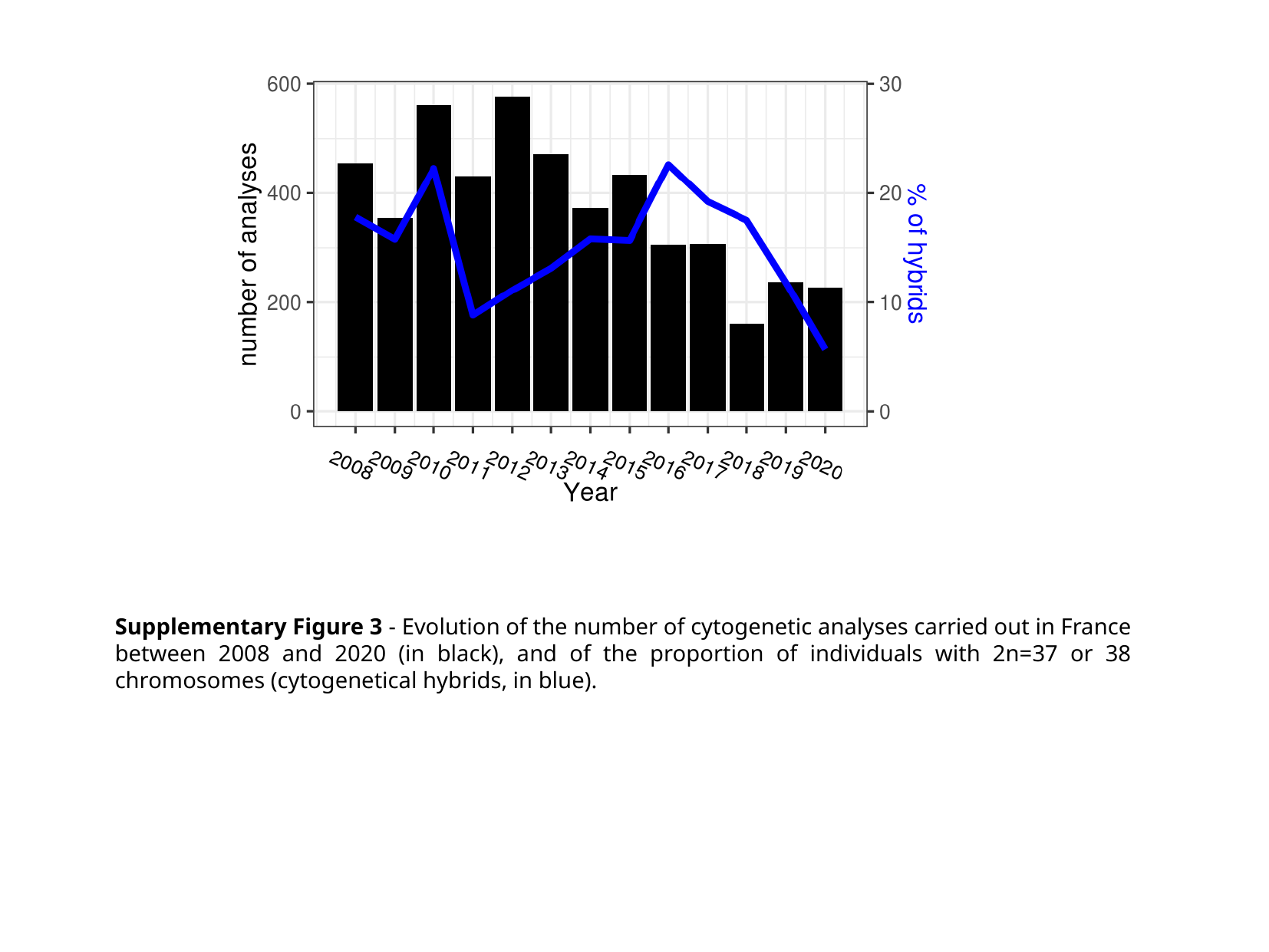

Supplementary Figure 3 - Evolution of the number of cytogenetic analyses carried out in France between 2008 and 2020 (in black), and of the proportion of individuals with 2n=37 or 38 chromosomes (cytogenetical hybrids, in blue).
