# Supplementary material for: Genome‐wide analysis of hybridization in wild boar populations reveals adaptive introgression from domestic pig
Source: Evol Appl. 2022 Jul 2;15(7):1115–28. doi: 10.1111/eva.13432 (PMC9309462; doi:10.1111/eva.13432)
Supplement: Supplementary file 4 — Figure S4 [file EVA-15-1115-s009.pptx]

## Slide 1
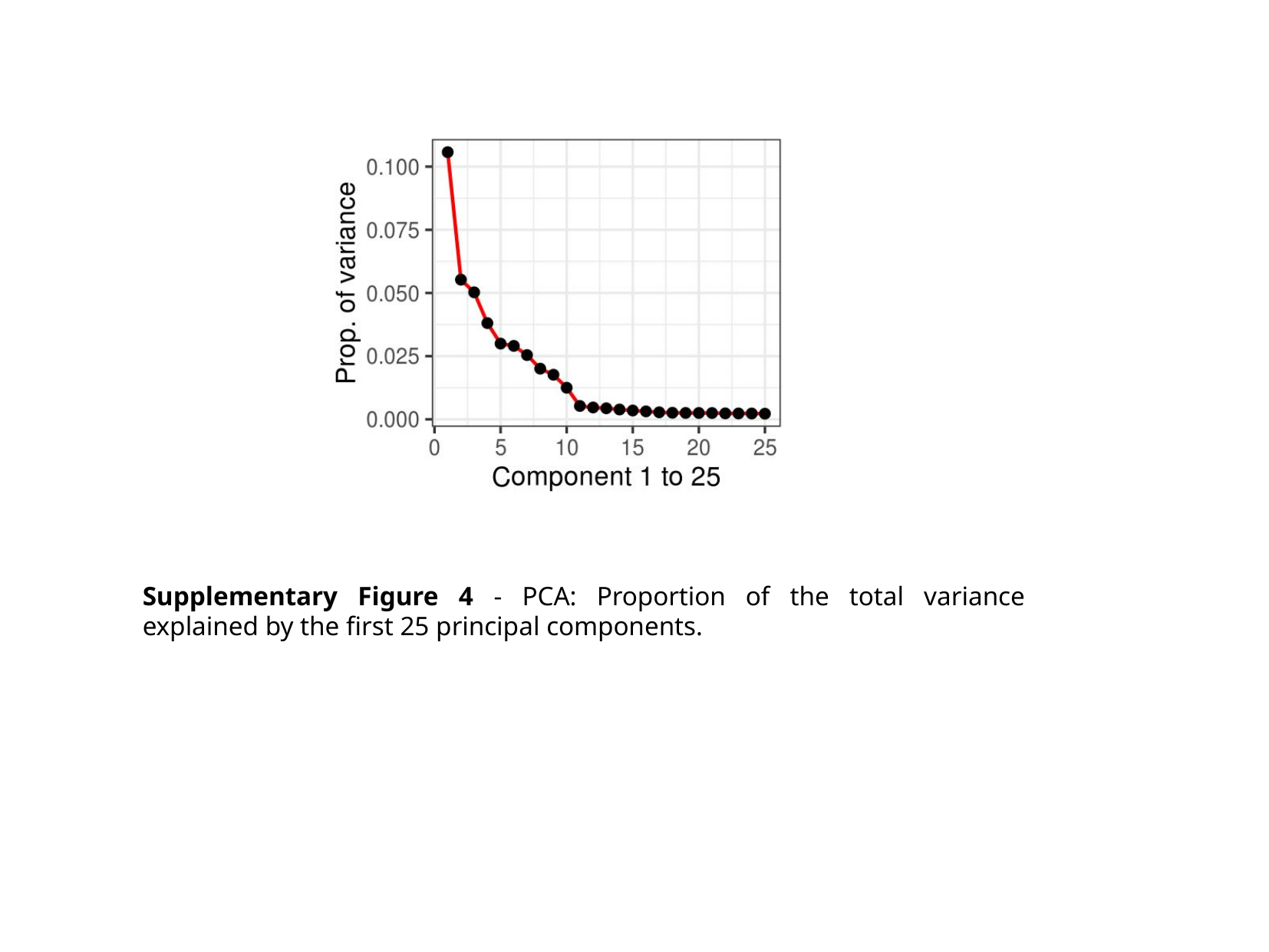

Supplementary Figure 4 - PCA: Proportion of the total variance explained by the first 25 principal components.
