# Supplementary material for: Genome‐wide analysis of hybridization in wild boar populations reveals adaptive introgression from domestic pig
Source: Evol Appl. 2022 Jul 2;15(7):1115–28. doi: 10.1111/eva.13432 (PMC9309462; doi:10.1111/eva.13432)
Supplement: Supplementary file 5 — Figure S5 [file EVA-15-1115-s005.pptx]

## Slide 1
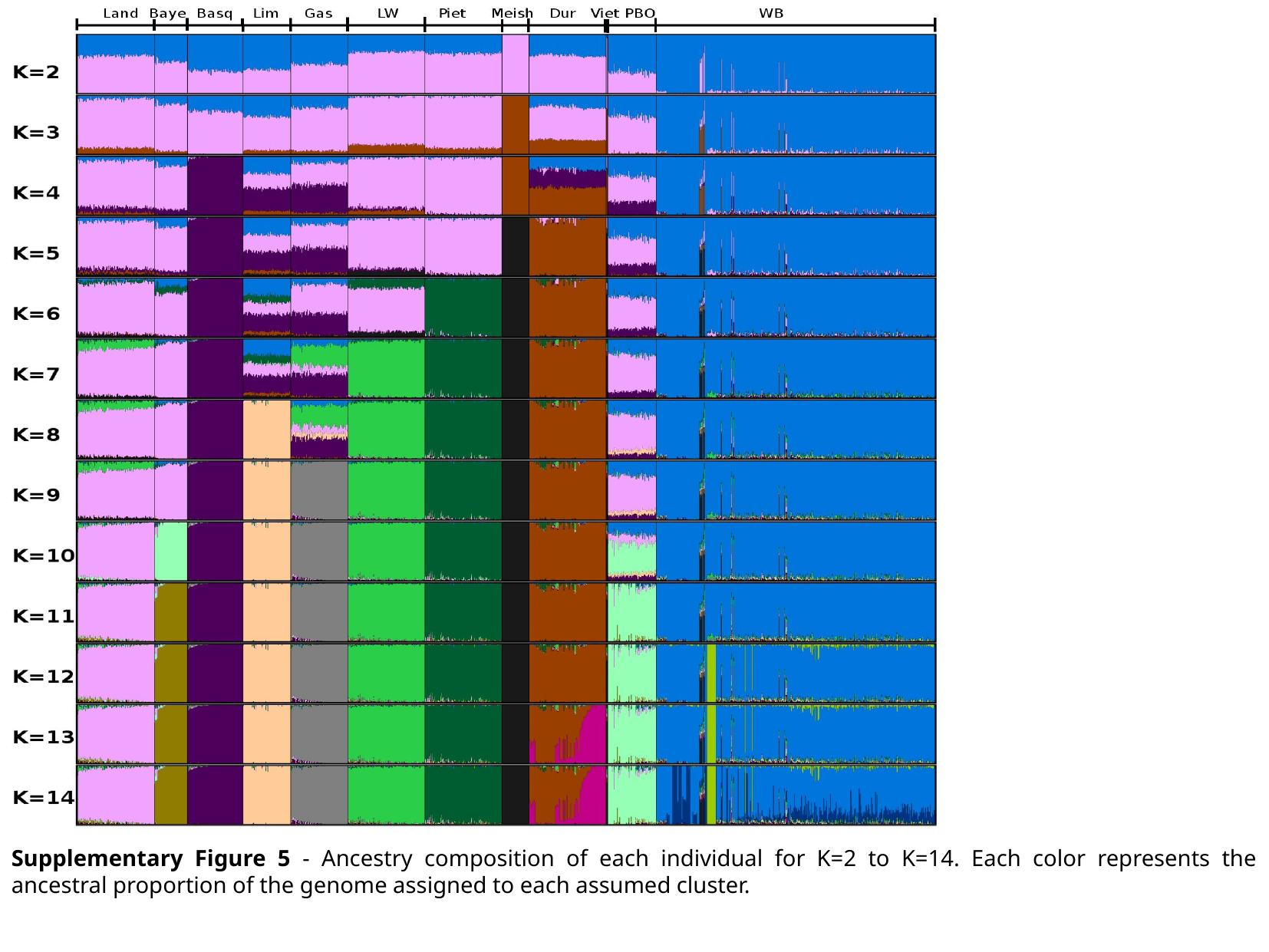

Supplementary Figure 5 - Ancestry composition of each individual for K=2 to K=14. Each color represents the ancestral proportion of the genome assigned to each assumed cluster.
