# Supplementary material for: Genome‐wide analysis of hybridization in wild boar populations reveals adaptive introgression from domestic pig
Source: Evol Appl. 2022 Jul 2;15(7):1115–28. doi: 10.1111/eva.13432 (PMC9309462; doi:10.1111/eva.13432)
Supplement: Supplementary file 7 — Figure S7 [file EVA-15-1115-s003.pptx]

## Slide 1
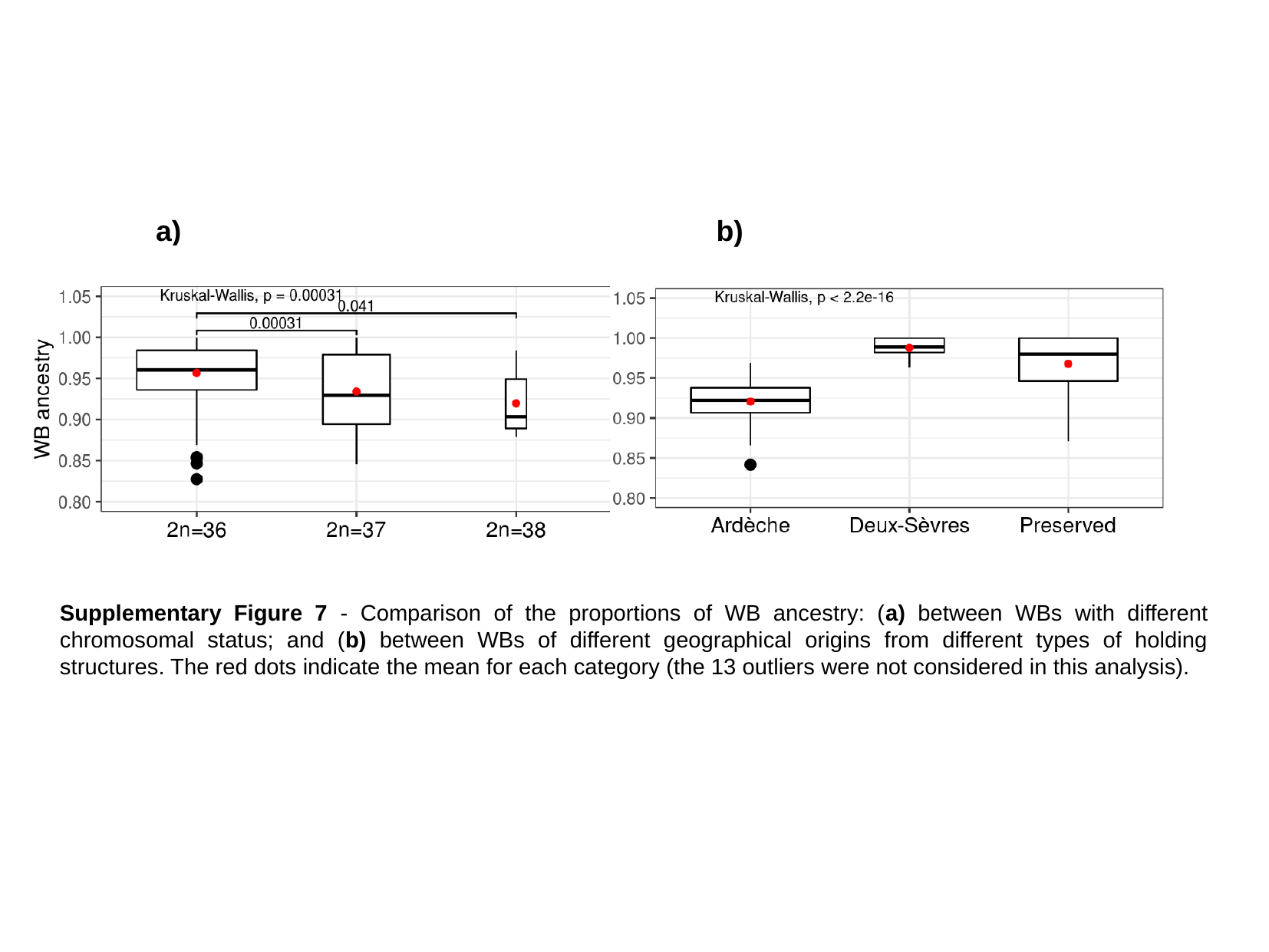

b)
a)
Supplementary Figure 7 - Comparison of the proportions of WB ancestry: (a) between WBs with different chromosomal status; and (b) between WBs of different geographical origins from different types of holding structures. The red dots indicate the mean for each category (the 13 outliers were not considered in this analysis).
