# Supplementary material for: Genome‐wide analysis of hybridization in wild boar populations reveals adaptive introgression from domestic pig
Source: Evol Appl. 2022 Jul 2;15(7):1115–28. doi: 10.1111/eva.13432 (PMC9309462; doi:10.1111/eva.13432)
Supplement: Supplementary file 8 — Figure S8 [file EVA-15-1115-s007.pptx]

## Slide 1
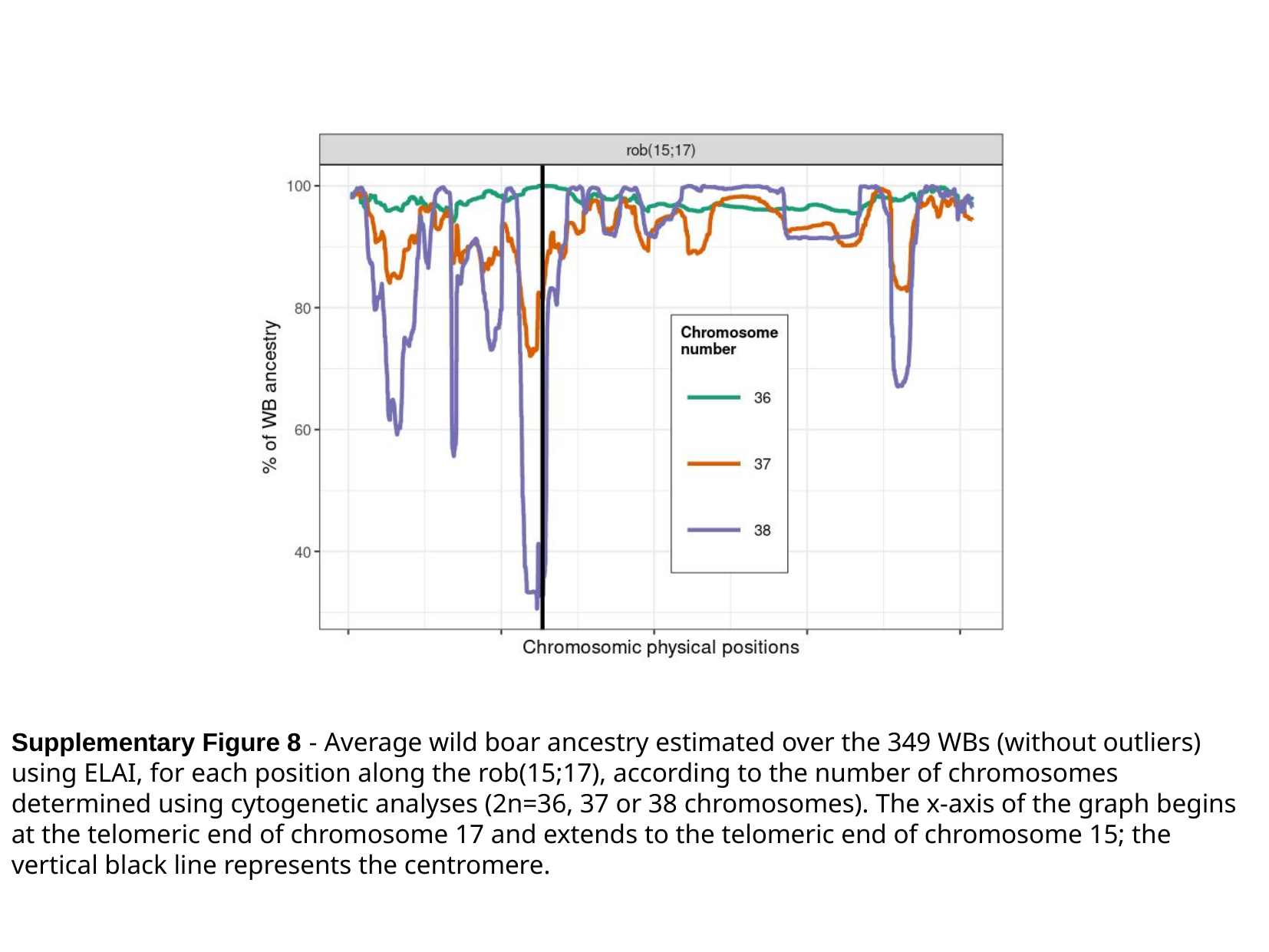

Supplementary Figure 8 - Average wild boar ancestry estimated over the 349 WBs (without outliers) using ELAI, for each position along the rob(15;17), according to the number of chromosomes determined using cytogenetic analyses (2n=36, 37 or 38 chromosomes). The x-axis of the graph begins at the telomeric end of chromosome 17 and extends to the telomeric end of chromosome 15; the vertical black line represents the centromere.
