# Supplementary material for: Genome‐wide analysis of hybridization in wild boar populations reveals adaptive introgression from domestic pig
Source: Evol Appl. 2022 Jul 2;15(7):1115–28. doi: 10.1111/eva.13432 (PMC9309462; doi:10.1111/eva.13432)
Supplement: Supplementary file 9 — Table S1 [file EVA-15-1115-s010.pptx]

## Slide 1
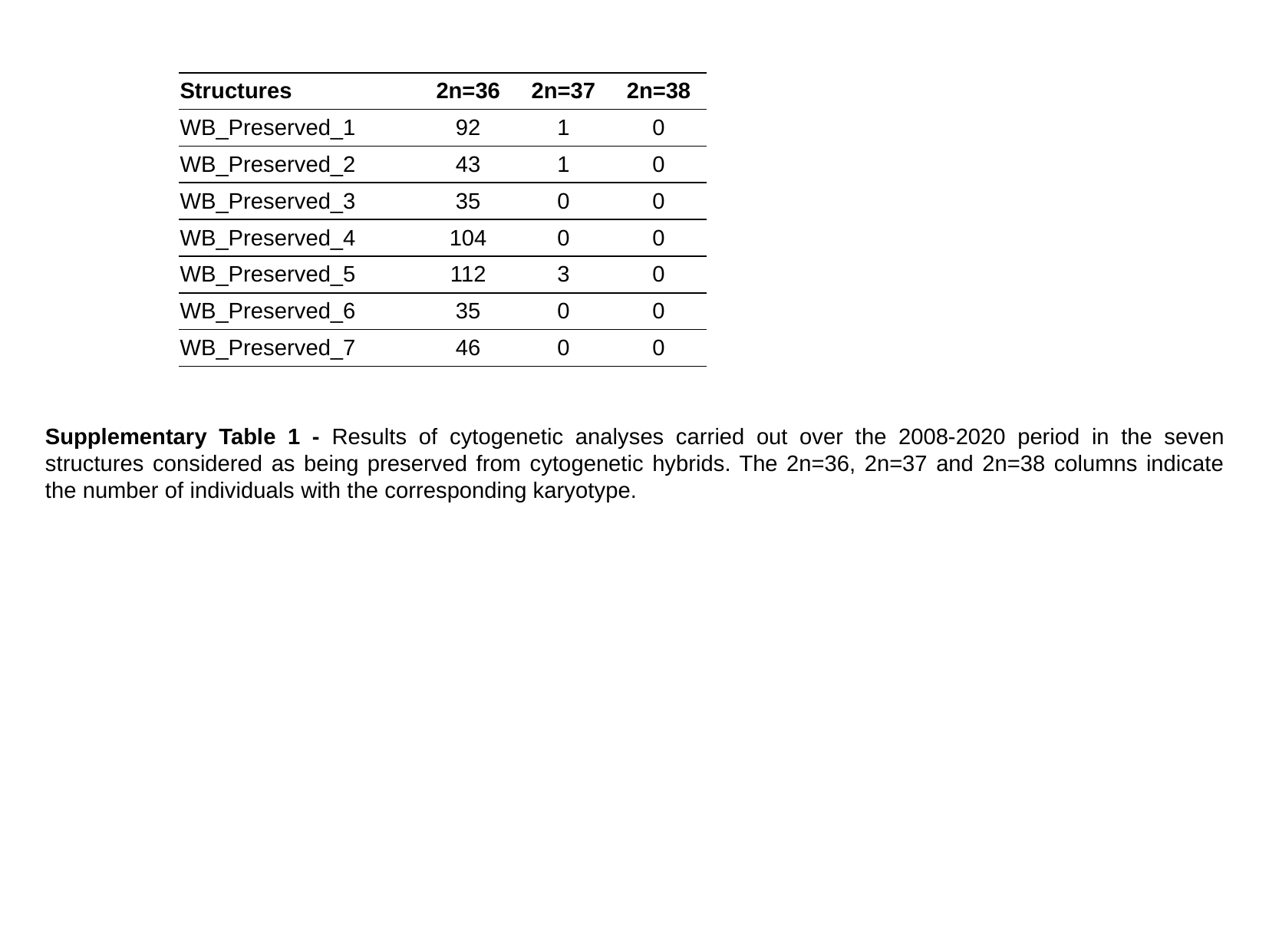

| Structures | 2n=36 | 2n=37 | 2n=38 |
| --- | --- | --- | --- |
| WB\_Preserved\_1 | 92 | 1 | 0 |
| WB\_Preserved\_2 | 43 | 1 | 0 |
| WB\_Preserved\_3 | 35 | 0 | 0 |
| WB\_Preserved\_4 | 104 | 0 | 0 |
| WB\_Preserved\_5 | 112 | 3 | 0 |
| WB\_Preserved\_6 | 35 | 0 | 0 |
| WB\_Preserved\_7 | 46 | 0 | 0 |
Supplementary Table 1 - Results of cytogenetic analyses carried out over the 2008-2020 period in the seven structures considered as being preserved from cytogenetic hybrids. The 2n=36, 2n=37 and 2n=38 columns indicate the number of individuals with the corresponding karyotype.
