# Supplementary material for: Genome‐wide analysis of hybridization in wild boar populations reveals adaptive introgression from domestic pig
Source: Evol Appl. 2022 Jul 2;15(7):1115–28. doi: 10.1111/eva.13432 (PMC9309462; doi:10.1111/eva.13432)
Supplement: Supplementary file 10 — Table S2 [file EVA-15-1115-s001.pptx]

## Slide 1
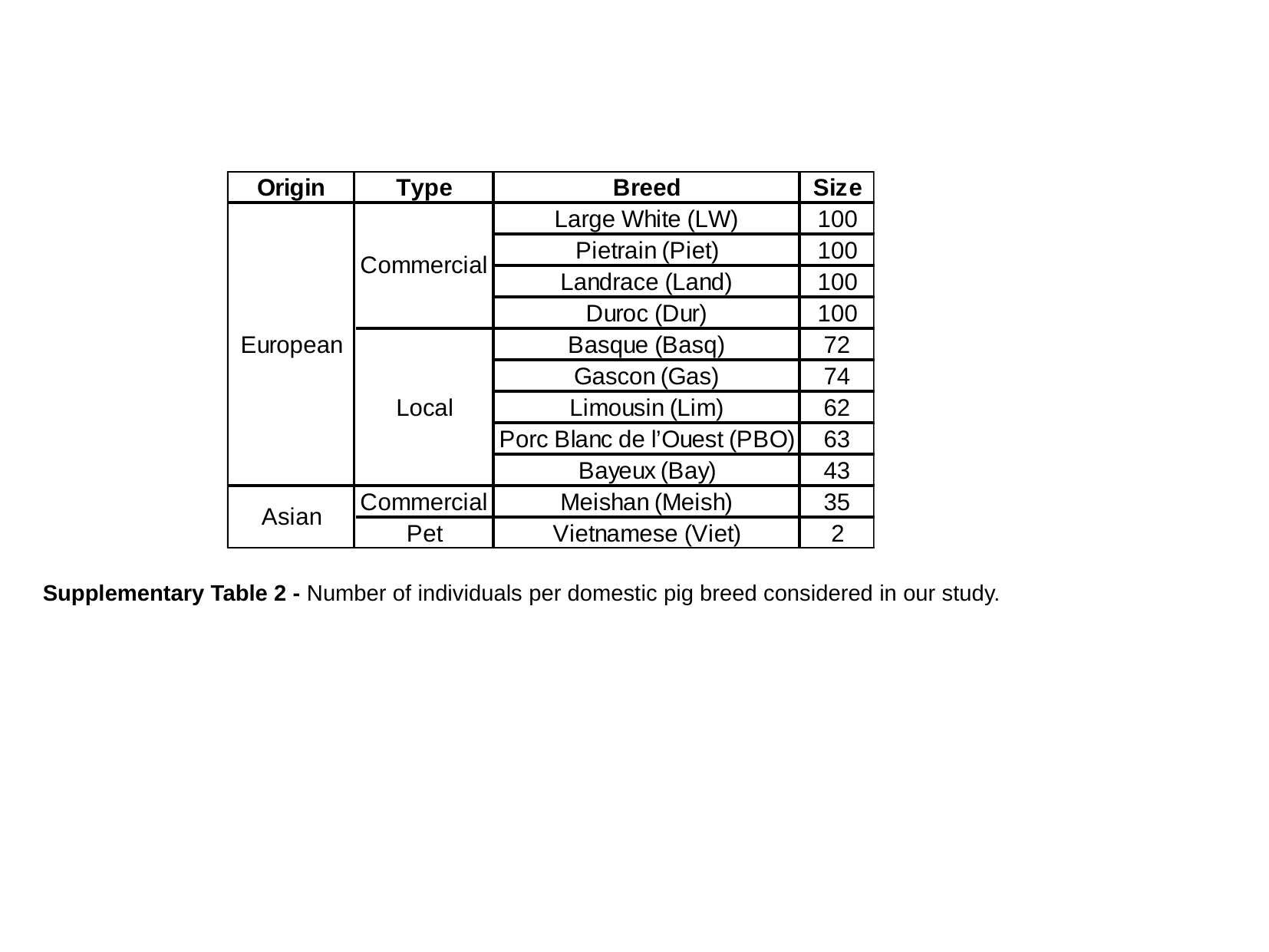

Supplementary Table 2 - Number of individuals per domestic pig breed considered in our study.
